# The family Stratiomyidae in Egypt and Saudi Arabia (Diptera: Stratiomyoidea)

**DOI:** 10.3897/BDJ.9.e64212

**Published:** 2021-03-22

**Authors:** Magdi El-Hawagry, Hathal Mohammed Al Dhafer, Mahmoud Abdel-Dayem, Martin Hauser

**Affiliations:** 1 Entomology Department, Faculty of Science, Cairo University, Giza, Egypt Entomology Department, Faculty of Science, Cairo University Giza Egypt; 2 King Saud University, College of Food and Agriculture Sciences, Riyadh, Saudi Arabia King Saud University, College of Food and Agriculture Sciences Riyadh Saudi Arabia; 3 California Department of Food & Agriculture, Sacramento, United States of America California Department of Food & Agriculture Sacramento United States of America

**Keywords:** soldier-flies, local distribution, dates of collection, new records

## Abstract

**Background:**

This study systematically catalogues all known taxa of the family Stratiomyidae in Egypt and Saudi Arabia. It is one in a series of planned studies aiming to catalogue the whole order in both countries.

**New information:**

Twenty species, belonging to seven genera and three subfamilies (Pachygastrinae, Stratiomyinae and Nemotelinae), are treated. One of these genera, *Oplodontha* and two species, *Oplodontha pulchriceps* Loew and *Oxycera turcica* Üstüner & Hasbenli, are recorded herein for the first time from Saudi Arabia. A lectotype for *Nemotelus matrouhensis* Mohammad et al., 2009 is designated. An updated classification, synonymies, type localities, world and local distributions, dates of collection and some coloured photographs are provided.

## Introduction

The Stratiomyidae is a lower brachycerous Dipteran family, including more than 2650 species classified in 375 genera and 12 subfamilies worldwide (Woodley 2001). Flies of the family Stratiomyidae, commonly known as soldier-flies, are small to large, ranging from 2 to 28 mm in length, highly varied in shape and colouration, with wings hyaline to variously patterned or infuscated and some species likely mimic wasps and bees in their flight and body colour (Hauser 2008, Hauser et al. 2017b). These flies can be easily distinguished as adults by the following characters: radial veins grouped together anteriorly, ending before tip of the wing; costal vein (C) usually ending well before wing apex and discal cell (d) short, usually forming distinct short, often squarish cell at middle of the wing (Marshall et al. 2017).

Adult soldier-flies can be found sitting on foliage in sunny locations in damp forest habitats, near water or boggy areas and some flies can be found frequenting flowers, particularly those of the subfamily Stratiomyinae and some of the subfamily Clitellariinae (James 1981, Khaghaninia et al. 2015, Hauser et al. 2017b).

Immature stages of soldier-flies can be found in a variety of habitats. Larvae of the subfamilies Beridinae, Clitellariinae and Sarginae, which have not been represented in Egypt or Saudi Arabia, are usually associated with decaying organic matter, such as leaf litter and rotting fruits. However, larvae of the subfamilies Nemotelinae and Stratiomyinae are aquatic, occurring in slow moving or standing water in rivers, ponds, seepage areas and other transient water sources, with those of the genus *Nemotelus* Geoffroy, 1762 being remarkably tolerant to high levels of salinity. Larvae of the subfamily Pachygastrinae can be found under the bark of fallen trees (Rozkošný 1983, Hauser 2008, Hauser et al. 2017b).

Of the 12 subfamilies, seven are recorded in the Palaearctic Region with about 430 species (Woodley 2001). Of these, only three subfamilies (Pachygastrinae, Stratiomyinae and Nemotelinae) have been represented in Saudi Arabia and/or Egypt by seven genera comprising 20 species. One of these genera, *Oplodontha* and two species, *Oxycera turcica* Üstüner & Hasbenli and *Oplodontha pulchriceps* Loew, are recorded herein for the first time from Saudi Arabia. This is not surprising as no previous faunistic or systematic studies on the Stratiomyidae have been carried out in Saudi Arabia and only two species have been recorded from Saudi Arabia amidst two comprehensive checklists of the Saudi Arabian Diptera: El-Hawagry et al. (2017) [*Aspidacantha atra* Kertesz] and Abu-Zoherah et al. (1993) [*Stratiomys deserticolor* Lindner]. We think the overall number of Saudi Arabian taxa treated in the present study is still low and does not represent the real fauna of Stratiomyidae in this country and an extensive faunistic and systematic study on the Saudi Arabian Stratiomyidae is required. On the other hand, some previous studies have been carried out in Egypt to list the Stratiomyidae species (Steyskal and El-Bialy 1967) or to study the fauna and/or taxonomy of the family (Lindner 1930, Badrawy 2006, Mohammad et al. 2009), in addition to some miscellaneous studies which described some new species from Egypt (Olivier 1811, Becker 1902, Wiedemann 1830, Lindner 1925, Lindner 1930, Lindner 1937, Lindner 1974).

**Figure 1.**
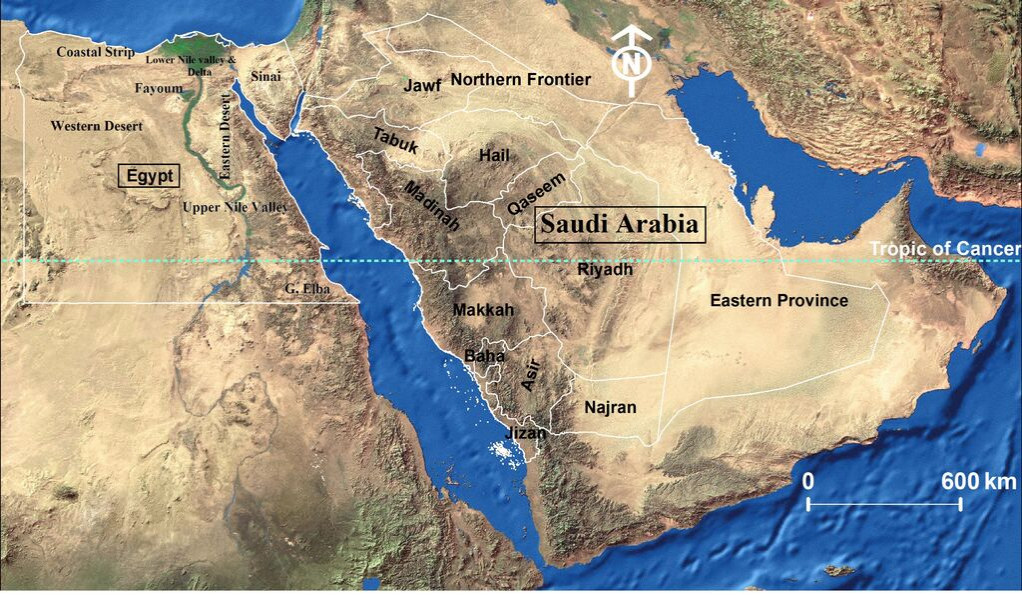
A satellite map of Egypt and Saudi Arabia.

Egypt and Saudi Arabia are two neighbouring countries in the Middle East, separated by the Gulf of Aqaba and the Red Sea (Fig. 1). Egypt is a transcontinental country as its majority is located in the north-eastern corner of Africa, while its north-eastern extremity, Sinai Peninsula, is located in the south-western corner of Asia. On the other hand, Saudi Arabia is wholly located in the south-western corner of Asia. Both Egypt and Saudi Arabia are biogeographically located at the junction of the Palaearctic and the Afrotropical Regions. The faunal affiliation of the two countries is mainly Palaearctic. Exceptions are Gebel Elba, the south-eastern triangle of Egypt and the southwestern part of Saudi Arabia, south to the Tropic of Cancer, which are considered as having an Afrotropical faunal affiliation (Wallace 1876, Hölzel 1998, El-Hawagry and Gilbert 2014, El-Hawagry et al. 2017).

This study is one in a series of taxonomic studies on different Egyptian and Saudi Arabian dipteran taxa aiming to catalogue the whole order Diptera in the two countries (EL-Hawagry 2015, Al Dhafer and El-Hawagry 2016, El-Hawagry 2017, EL-Hawagry 2018, El-Hawagry et al. 2018, El-Hawagry and Al Dhafer 2019, El-Hawagry et al. 2019, El-Hawagry and Gilbert 2019, El-Hawagry et al. 2020b, El-Hawagry et al. 2020a, El-Hawagry et al. 2021, El-Hawagry and El-Azab 2019).

## Materials and methods

**Data sources**. Data of the present study are obtained from three main sources: 1. Specimens preserved in the Egyptian and Saudi Arabian insect collections and museums, namely: Efflatoun Bey’s collection, Cairo University, Egypt (EFC); Collection of Entomological Society of Egypt (ESEC) and King Saud University Museum of Arthropods, Saudi Arabia (KSMA). 2. Previous studies on the Stratiomyidae in Egypt and Saudi Arabia. 3. Specimens collected by the authors and their co-workers, especially from Saudi Arabia using both Malaise traps and aerial nets. A great deal of faunistic and taxonomic information, including type species, type localities, Old World synonymies, world and local distributions and collection dates were obtained from relevant literature as well. These sources are listed in the following subsections.

**Study area**. Egypt and Saudi Arabia, the study area, are two neighbouring countries in the Middle East, separated by the Red Sea and the Gulf of Aqaba. They constitute a part of the Great Desert Belt, mainly with an arid desert climate characterised by hot summer and a mild winter (Alsharhan et al. 2001, Nasser et al. 2019).

Ecologists divide Egypt into eight ecological zones: the Coastal Strip, Lower Nile Valley & Delta, Upper Nile Valley, Fayoum, Eastern Desert, Western Desert, Sinai and Gebel Elba (Fig. 1, El-Hawagry and Gilbert 2014). These ecological zones are adopted in the present study in the sections of localities and dates of collection. However, Saudi Arabia is not divided into ecological zones, so ecologists usually adopt the administrative divisions instead. These divisions (also called emirates, regions or provinces) are adopted in the present study, namely: Makkah, Riyadh, Eastern Province, Asir, Jazan, Al-Madinah, Al-Qaseem, Tabuk, Hail, Najran, Al-Jawf, Al-Baha and Northern Frontier (El-Hawagry and Al Dhafer 2019).

**Classification.** The classification and arrangement of taxa in the present study basically follows that used in Woodley (2001) and subsequent updates by Hauser et al. (2017a).

**World distribution**. Sources of world distribution of each stratiomyid species are given between square brackets at the end of the list of countries.

**Local distribution and dates of collection**. Localities within each Egyptian ecological zone or Saudi Arabian administrative region are arranged alphabetically and written after a colon following each zone or region followed by the dates of collection between parentheses; for example, "Coastal Strip: Abu-Kir, Cleopatra, Dekhela (April to September)" and “Tabuk: Tabuk City (May)”. Sources for this distribution are given between square brackets at the end of the section. Coordinates of all Egyptian and Saudi Arabian localities of the family Stratiomyidae are listed (Table 1). Distribution maps of species were made using SimpleMappr (Shorthouse 2010).

Abbreviations used:

- **AF**, Afrotropical
- **CSCA**, California State Collection of Arthropods, Sacramento, California, USA
- **EFC**, Efflatoun Bey’s collection, Department of Entomology, Faculty of Science, Cairo University, Egypt
- **ESEC**, Collection of Entomological Society of Egypt, Cairo, Egypt
- **KSMA**, King Saud University Museum of Arthropods, Riyadh, Saudi Arabia
- **MCCB**, Museum of Community College, Al-Baha University, Saudi Arabia
- **OR**, Oriental
- **PA**, Palearctic

## Checklists

### The Catalogue

#### Stratiomyidae

931D5370-5409-5F83-A6D0-7DB38E012480

#### Stratiomyiinae

33B098FD-8763-51CC-A118-4E3315DFF0E8

#### Aspidacantha

Kertsz, 1916

205E6D2F-CE54-5634-BC1A-FC3E58007A8F

https://www.gbif.org/species/1578168

Aspidacantha Kertész, 1916: 154. Type species: *Aspidacantha atra* Kertész, by original designation.

#### Aspidacantha atra

Kertsz, 1916

081AA6FB-0EBC-56F1-904D-113D9976E520

https://www.gbif.org/species/1578171

Aspidacantha atra Kertész, 1916: 155. Type locality: Eritrea (Assab) [Assab belongs now to Eritrea not to Ethiopia as written in world catalogues].

##### Materials

- **Type status:** Other material. **Occurrence:** recordedBy: H. Pohl; sex: 1 female; lifeStage: Adult; **Taxon:** taxonID: https://www.gbif.org/species/1578171; scientificName: Aspidacantha atra; **Location:** country: Egypt; locality: Hurghada; decimalLatitude: 27.23370; decimalLongitude: 33.8256; **Identification:** identifiedBy: M. El-Hawagry & M. Hauser; dateIdentified: 2020-2021; **Event:** samplingProtocol: Light trap; eventDate: 9/11-22/1994; **Record Level:** institutionCode: CSCA
- **Type status:** Other material. **Occurrence:** recordedBy: Aldhafer H.M. et al.; lifeStage: Adult; **Taxon:** taxonID: https://www.gbif.org/species/1578171; scientificName: Aspidacantha atra; **Location:** country: Saudi Arabia; locality: Raydah; decimalLatitude: 18.20525; decimalLongitude: 42.4101; **Identification:** identifiedBy: M. El-Hawagry & M. Hauser; dateIdentified: 2020-2021; **Event:** samplingProtocol: Sweeping; eventDate: 06-07-2014; **Record Level:** institutionCode: KSMA
- **Type status:** Other material. **Occurrence:** recordedBy: Aldhafer H.M. et al.; lifeStage: Adult; **Taxon:** taxonID: https://www.gbif.org/species/1578171; scientificName: Aspidacantha atra; **Location:** country: Saudi Arabia; locality: Raydah; decimalLatitude: 18.201583; decimalLongitude: 42.4089; **Identification:** identifiedBy: M. El-Hawagry & M. Hauser; dateIdentified: 2020-2021; **Event:** samplingProtocol: Light trap; eventDate: 02-21-2014; **Record Level:** institutionCode: KSMA
- **Type status:** Other material. **Occurrence:** recordedBy: Aldhafer H.M. et al.; lifeStage: Adult; **Taxon:** taxonID: https://www.gbif.org/species/1578171; scientificName: Aspidacantha atra; **Location:** country: Saudi Arabia; locality: Raydah; decimalLatitude: 18.201583; decimalLongitude: 42.4089; **Identification:** identifiedBy: M. El-Hawagry & M. Hauser; dateIdentified: 2020-2021; **Event:** samplingProtocol: Light trap; eventDate: 07-31-2015; **Record Level:** institutionCode: KSMA
- **Type status:** Other material. **Occurrence:** recordedBy: Aldhafer H.M. et al.; lifeStage: Adult; **Taxon:** taxonID: https://www.gbif.org/species/1578171; scientificName: Aspidacantha atra; **Location:** country: Saudi Arabia; locality: Raydah; decimalLatitude: 18.20525; decimalLongitude: 42.4102; **Identification:** identifiedBy: M. El-Hawagry & M. Hauser; dateIdentified: 2020-2021; **Event:** samplingProtocol: Sweeping; eventDate: 09-05-2015; **Record Level:** institutionCode: KSMA
- **Type status:** Other material. **Occurrence:** recordedBy: Aldhafer H.M. et al.; lifeStage: Adult; **Taxon:** taxonID: https://www.gbif.org/species/1578171; scientificName: Aspidacantha atra; **Location:** country: Saudi Arabia; locality: Raydah; decimalLatitude: 18.201583; decimalLongitude: 42.4089; **Identification:** identifiedBy: M. El-Hawagry & M. Hauser; dateIdentified: 2020-2021; **Event:** samplingProtocol: Light trap; eventDate: 09-05-2015; **Record Level:** institutionCode: KSMA
- **Type status:** Other material. **Occurrence:** recordedBy: Aldhafer H.M. et al.; lifeStage: Adult; **Taxon:** taxonID: https://www.gbif.org/species/1578171; scientificName: Aspidacantha atra; **Location:** country: Saudi Arabia; locality: Raydah; decimalLatitude: 18.201583; decimalLongitude: 42.4089; **Identification:** identifiedBy: M. El-Hawagry & M. Hauser; dateIdentified: 2020-2021; **Event:** samplingProtocol: Malaise trap; eventDate: 09-05-2015; **Record Level:** institutionCode: KSMA
- **Type status:** Other material. **Occurrence:** recordedBy: Aldhafer H.M. et al.; lifeStage: Adult; **Taxon:** taxonID: https://www.gbif.org/species/1578171; scientificName: Aspidacantha atra; **Location:** country: Saudi Arabia; locality: Raydah; decimalLatitude: 18.204417; decimalLongitude: 42.4124; **Identification:** identifiedBy: M. El-Hawagry & M. Hauser; dateIdentified: 2020-2021; **Event:** samplingProtocol: Malaise trap; eventDate: 02-20-2014; **Record Level:** institutionCode: KSMA
- **Type status:** Other material. **Occurrence:** recordedBy: Aldhafer H.M. et al.; lifeStage: Adult; **Taxon:** taxonID: https://www.gbif.org/species/1578171; scientificName: Aspidacantha atra; **Location:** country: Saudi Arabia; locality: Raydah; decimalLatitude: 18.20525; decimalLongitude: 42.4101; **Identification:** identifiedBy: M. El-Hawagry & M. Hauser; dateIdentified: 2020-2021; **Event:** samplingProtocol: Malaise trap; eventDate: 06-07-2014; **Record Level:** institutionCode: KSMA

##### Distribution

AF: Democratic Republic of Congo, Eritrea, Ethiopia, Saudi Arabia [as “south western part”], Tanzania, Uganda, United Arab Emirates, Zimbabwe. PA: Egypt, Israel, Russia, Turkmenistan. [Sources: Lindner (1936), Woodley (2001), Hauser (2008) and El-Hawagry et al. (2017)]

**Figure 2.**
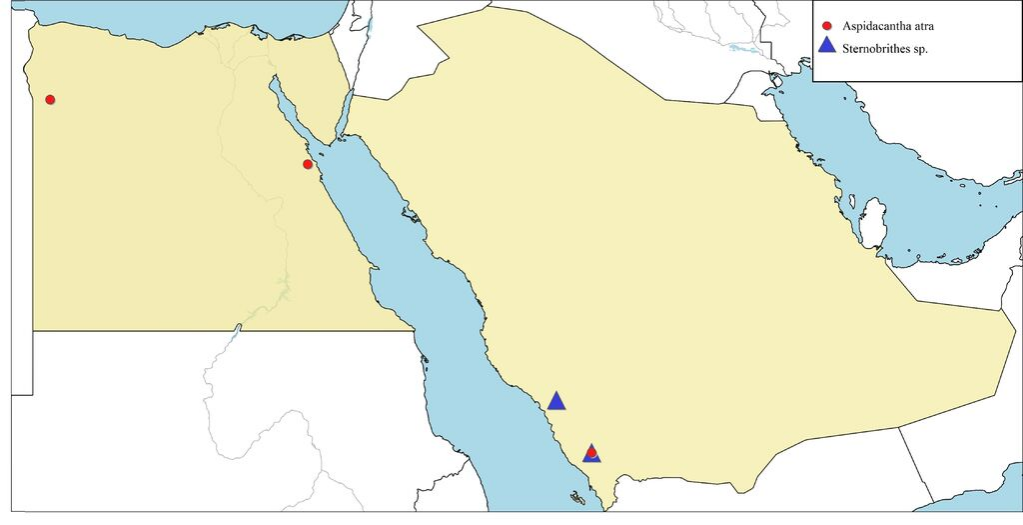
Distribution map of *Aspidacantha atra* Kertész and *Sternobrithes* sp.

**Local distribution and dates of collection** (Fig. 2): EGYPT: Eastern Desert: Hurghada (September). Western Desert: Siwa Oasis (August). [Sources: Lindner (1930) and museum material]. SAUDI ARABIA: Asir: Raydah Nature Reserve (February to December). [Sources: El-Hawagry et al. (2017) and collected material]

#### Sternobrithes

Loew, 1857

6900F789-4AEF-5661-9FA8-AA1793F98088

https://www.gbif.org/species/1576881

Sternobrithes Loew, 1857: 264. Type species *Sternobrithes tumidus* Loew, by monotypy.

#### Sternobrithes sp.

A5F755C2-AE97-546D-A32C-99D07F4AA224

https://www.gbif.org/species/1576881

##### Materials

- **Type status:** Other material. **Occurrence:** recordedBy: Aldhafer H.M. et al.; lifeStage: Adult; **Taxon:** taxonID: https://www.gbif.org/species/1576881; scientificName: *Sternobrithes* sp.; **Location:** country: Saudi Arabia; locality: Shada; decimalLatitude: 19.8429; decimalLongitude: 41.3115; **Identification:** identifiedBy: M. El-Hawagry & M. Hauser; dateIdentified: 2020-2021; **Event:** samplingProtocol: Malaise trap; eventDate: 11-15-2015; **Record Level:** institutionCode: KSMA
- **Type status:** Other material. **Occurrence:** recordedBy: Aldhafer H.M. et al.; lifeStage: Adult; **Taxon:** taxonID: https://www.gbif.org/species/1576881; scientificName: *Sternobrithes* sp.; **Location:** country: Saudi Arabia; locality: Shada; decimalLatitude: 19.8429; decimalLongitude: 41.3115; **Identification:** identifiedBy: M. El-Hawagry & M. Hauser; dateIdentified: 2020-2021; **Event:** samplingProtocol: Malaise trap; eventDate: 05-05-2015; **Record Level:** institutionCode: KSMA
- **Type status:** Other material. **Occurrence:** recordedBy: Aldhafer H.M. et al.; lifeStage: Adult; **Taxon:** taxonID: https://www.gbif.org/species/1576881; scientificName: *Sternobrithes* sp.; **Location:** country: Saudi Arabia; locality: Shada; decimalLatitude: 19.8388; decimalLongitude: 41.3101; **Identification:** identifiedBy: M. El-Hawagry & M. Hauser; dateIdentified: 2020-2021; **Event:** samplingProtocol: Malaise trap; eventDate: 11-15-2015; **Record Level:** institutionCode: KSMA
- **Type status:** Other material. **Occurrence:** recordedBy: Aldhafer H.M. et al.; lifeStage: Adult; **Taxon:** taxonID: https://www.gbif.org/species/1576881; scientificName: *Sternobrithes* sp.; **Location:** country: Saudi Arabia; locality: Shada; decimalLatitude: 19.8388; decimalLongitude: 41.3101; **Identification:** identifiedBy: M. El-Hawagry & M. Hauser; dateIdentified: 2020-2021; **Event:** samplingProtocol: Light trap; eventDate: 09-02-2015; **Record Level:** institutionCode: KSMA

##### Distribution

The genus *Sternobrithes* is widely distributed all over the African continent (Botswana, Burundi, Cameroon, Cape Verde Islands, Democratic Republic of the Congo, Ethiopia, Ghana, Guinea, Ivory Coast, Kenya, Liberia, Malawi, Mozambique, Namibia, Nigeria, Senegal, Sierra Leone, Somalia, South Africa, Tanzania, Togo, Uganda and Zimbabwe. [Sources: Woodley (2001)]

**Distribution of *Sternobrithes* sp.**: AF: Kenya, Saudi Arabia [as “south western part”], Yemen.

**Local distribution and dates of collection** (Fig. 2): SAUDI ARABIA: Al-Baha: Jabal Shada al-A’la Nature Reserve (May to November). Asir: Raydah Nature Reserve (June). [Sources: El-Hawagry et al. (2017) and collected material]

##### Notes

**Figure 3.**
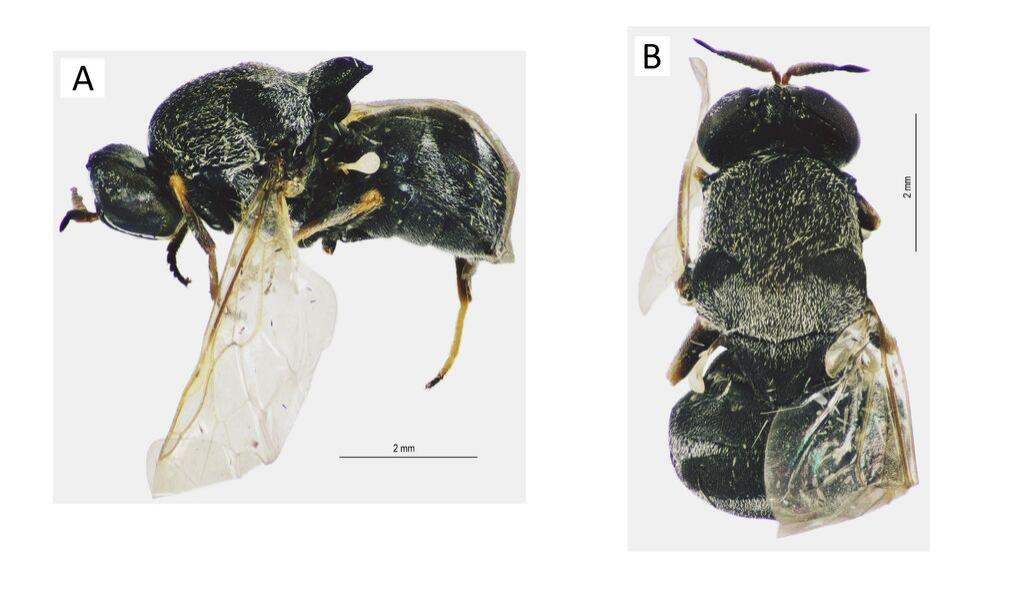
*Sternobrithes* sp.: **A.** female habitus, lateral; **B.** same, dorsal.

El-Hawagry et al. (2017) reported this species as *Sternobrithes* sp. and further research indicated that this is an undescribed species (Fig. 3). As we do not have a male specimen available, we are not yet describing this species here. According the generic key in Hauser et al. (2017b), the difference between *Argyrobrithes* and *Sternobrithes* is the colour of the last antennal segment, but further studies indicate that *Argyrobrithes* is characterised by the dichoptic male eyes and a very long last antennal segment. The two genera are widely distributed through Africa and the Oriental Region and a more thorough revision is needed to clarify the limits of the genera and which species should be included in each genus. Material of this undescribed species are known from Yemen and Kenya.

#### Stratiomyinae

E60C20CF-65A2-5F7A-ACC7-14B58E3F4B2B

#### Oxycerini

6E494BD5-9091-5FF6-BD63-9B9851B9821F

#### Oxycera

Meigen, 1803

4E5455B3-8E70-55AA-B7C3-DA5E5E3CD962

https://www.gbif.org/species/1577931

Oxycera Meigen, 1803: 265. Type species: *Musca hypoleon* Linnaeus [= *Musca trilineata* Linnaeus], by designation of Latreille (1810).

#### Oxycera orientalis

(Lindner, 1974)

68A7407C-36A4-5310-B2AE-27ACC7656E2C

https://www.gbif.org/species/5078177

Heraclina orientalis Lindner, 1974: 103. Type locality: Egypt (Sinai Mountains: Wadi Watir) [Wadi Watir is located in Sinai, Egypt, not in Israel as given in world catalogues].

##### Materials

- **Type status:** Other material. **Occurrence:** sex: 1 male; lifeStage: Adult; **Taxon:** taxonID: https://www.gbif.org/species/5078177; scientificName: Oxycera orientalis; **Location:** country: Egypt; locality: Wadi Watir; decimalLatitude: 29.02147; decimalLongitude: 34.6731; **Identification:** identifiedBy: M. El-Hawagry & M. Hauser; dateIdentified: 2020-2021; **Event:** samplingProtocol: Not given; eventDate: 09-06-1941; **Record Level:** institutionCode: EFC

##### Distribution

PA: Egypt. [Sources: Furth (1983), Woodley (2001) and Badrawy (2006)]

**Figure 4.**
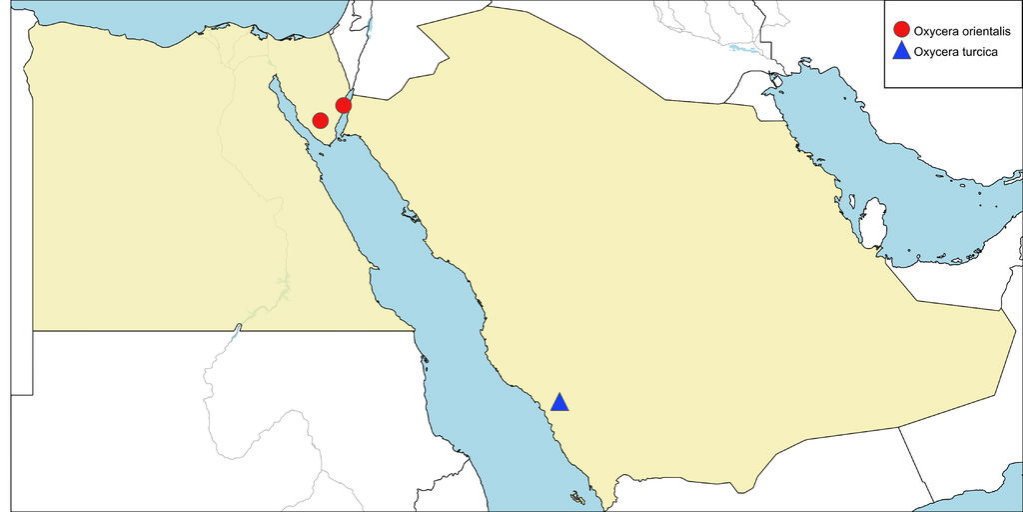
Distribution map of *Oxycera orientalis* (Lindner) and *Oxycera turcica* Üstüner & Hasbenli.

**Local distribution and dates of collection** (Fig. 4): EGYPT: Sinai: Wadi El-Lega, Wadi Watir (Ain Fortaga) (September to November). [Sources: original description (Lindner 1974) and museum material]

#### Oxycera turcica

Üstüner & Hasbenli, 2005

3D828CE0-4966-5794-9AB6-159D8DBE9D5A

https://www.gbif.org/species/5078139

Oxycera turcica Üstüner & Hasbenli, 2005: 163. Type locality: Turkey (Sivas: Sarkisla, Karacaören Village).

##### Materials

- **Type status:** Other material. **Occurrence:** recordedBy: El-Hawagry; lifeStage: Adult; **Taxon:** taxonID: https://www.gbif.org/species/5078139; scientificName: Oxycera turcica; **Location:** country: Saudi Arabia; locality: Al-Mekhwa; decimalLatitude: 19.798133; decimalLongitude: 41.4107; **Identification:** identifiedBy: M. El-Hawagry & M. Hauser; dateIdentified: 2020-2021; **Event:** samplingProtocol: Malaise trap; eventDate: 03-15-2012; **Record Level:** institutionCode: MCCB

##### Distribution

PA: Saudi Arabia, Turkey. [Sources: Woodley (2011) and collected material]

**Local distribution and dates of collection** (Fig. 4): SAUDI ARABIA: Al-Baha: Al-Mekhwa (March). [Source: collected material]

##### Notes

This species is recorded herein for the first time from Saudi Arabia.

#### Stratiomyini

EC102921-A038-53EC-80F2-0CF9A9A17449

#### Odontomyia

Meigen, 1803

E15F2AE8-41FF-5252-9A3E-00AA2DA9CAB8

https://www.gbif.org/species/1580809

Odontomyia Meigen, 1803: 265. Type species: *Musca hydroleon* Linnaeus, by designation of Westwood (1840).

#### Odontomyia angulata

(Panzer, 1798)

DC32212A-8253-58EF-B199-38CBA0008E8A

https://www.gbif.org/species/1581104

Stratiomys angulata Panzer, 1798: 19. Type locality: Germany (Nürnberg).
Stratiomys vulpina Panzer, 1798: 24. Type locality: Germany (Nürnberg).
Stratiomys hydropota Meigen, 1822: 147. Type locality: Europe.
Odontomyia latifaciata Macquart, 1834: 248. Type locality: France.
Stratiomys brevicornis Loew, 1840: 25. Type locality: Poland (Poznañ).
Stratiomys brevicornis Loew, 1840: 557. Type locality: Poland (Poznañ). Preoccupied, primary homonym of *Stratiomys brevicornis* Loew, 1840.
Stratiomys ruficornis Zetterstedt, 1842: 139. Type locality: Sweden (Gotland: Ejsta). Preoccupied, primary homonym of *Stratiomyia ruficornis* Macquart, 1838.
Odontomyia hydrophila Loew, 1846: 486. Type locality: Turkey (Makri) and Italy (Sicily: Siracusa).
Eulalia latifasciata Kertész, 1908: 74. Unjustified emendation of *Odontomyia latifaciata* Macquart.

##### Distribution

PA: Afghanistan, Albania, Algeria, Austria, Belgium, Bulgaria, China, Czech Republic, Denmark, Egypt, England, Estonia, Finland, France, Germany, Greece, Hungary, Iran, Israel, Italy including Sardinia, Kazakhstan, Morocco, Netherlands, Poland, Romania, Russia, Slovakia, Spain, Sweden, Switzerland, Turkey, UAE, former Yugoslavia. [Sources: Woodley (2001), Mason et al. (2009), Hauser (2014) and Yimlahi et al. (2017)]

**Local distribution and dates of collection**: Unknown.

##### Notes

This species was listed by Steyskal and El-Bialy (1967), Woodley (2001) and Badrawy (2006) as recorded from Egypt, but no specimens have been collected or preserved in Egyptian museums to confirm these records.

#### Odontomyia megacephala

Olivier, 1811

2D7491E4-4962-5031-9DA8-1A82AB6A75FD

https://www.gbif.org/species/7863197

Odontomyia megacephala Olivier, 1811: 432. Type locality: Egypt (borders of the Nile [as “bords du Nil”]).

##### Materials

- **Type status:** Other material. **Occurrence:** recordedBy: Efflatoun; lifeStage: Adult; **Taxon:** taxonID: https://www.gbif.org/species/7863197; scientificName: Odontomyia megacephala; **Location:** country: Egypt; locality: Moweileh; decimalLatitude: 30.3924; decimalLongitude: 34.1412; **Identification:** identifiedBy: M. El-Hawagry & M. Hauser; dateIdentified: 2020-2021; **Event:** samplingProtocol: Not given; eventDate: Sept.1924; **Record Level:** institutionCode: ESEC
- **Type status:** Other material. **Occurrence:** recordedBy: Efflatoun; lifeStage: Adult; **Taxon:** taxonID: https://www.gbif.org/species/7863197; scientificName: Odontomyia megacephala; **Location:** country: Egypt; locality: Kosseima; decimalLatitude: 30.90307; decimalLongitude: 34.3836; **Identification:** identifiedBy: M. El-Hawagry & M. Hauser; dateIdentified: 2020-2021; **Event:** samplingProtocol: Not given; eventDate: 09-10-1924; **Record Level:** institutionCode: ESEC
- **Type status:** Other material. **Occurrence:** recordedBy: Efflatoun; lifeStage: Adult; **Taxon:** taxonID: https://www.gbif.org/species/7863197; scientificName: Odontomyia megacephala; **Location:** country: Egypt; locality: Kharga Oasis; decimalLatitude: 25.25; decimalLongitude: 30.5833; **Identification:** identifiedBy: M. El-Hawagry & M. Hauser; dateIdentified: 2020-2021; **Event:** samplingProtocol: Not given; eventDate: Oct.1924; **Record Level:** institutionCode: ESEC
- **Type status:** Other material. **Occurrence:** recordedBy: Efflatoun; lifeStage: Adult; **Taxon:** taxonID: https://www.gbif.org/species/7863197; scientificName: Odontomyia megacephala; **Location:** country: Egypt; locality: Fayoum; decimalLatitude: 29.32061; decimalLongitude: 30.818; **Identification:** identifiedBy: M. El-Hawagry & M. Hauser; dateIdentified: 2020-2021; **Event:** samplingProtocol: Not given; eventDate: Sept.1924; **Record Level:** institutionCode: ESEC
- **Type status:** Other material. **Occurrence:** recordedBy: Efflatoun; sex: 1 female; lifeStage: Adult; **Taxon:** taxonID: https://www.gbif.org/species/7863197; scientificName: Odontomyia megacephala; **Location:** country: Egypt; locality: Alexandria; decimalLatitude: 31.2129; decimalLongitude: 29.9726; **Identification:** identifiedBy: M. El-Hawagry & M. Hauser; dateIdentified: 2020-2021; **Event:** samplingProtocol: Not given; eventDate: 07-04-1920; **Record Level:** institutionCode: EFC
- **Type status:** Other material. **Occurrence:** recordedBy: Efflatoun; sex: 1 female; lifeStage: Adult; **Taxon:** taxonID: https://www.gbif.org/species/7863197; scientificName: Odontomyia megacephala; **Location:** country: Egypt; locality: Nuzha; decimalLatitude: 31.2001; decimalLongitude: 29.9436; **Identification:** identifiedBy: M. El-Hawagry & M. Hauser; dateIdentified: 2020-2021; **Event:** samplingProtocol: Not given; eventDate: 05-07-1921; **Record Level:** institutionCode: EFC
- **Type status:** Other material. **Occurrence:** recordedBy: Efflatoun; sex: 1 male; lifeStage: Adult; **Taxon:** taxonID: https://www.gbif.org/species/7863197; scientificName: Odontomyia megacephala; **Location:** country: Egypt; locality: Nuzha; decimalLatitude: 31.2001; decimalLongitude: 29.9436; **Identification:** identifiedBy: M. El-Hawagry & M. Hauser; dateIdentified: 2020-2021; **Event:** samplingProtocol: Not given; eventDate: 07-18-1921; **Record Level:** institutionCode: EFC
- **Type status:** Other material. **Occurrence:** recordedBy: Efflatoun; sex: 2 females; lifeStage: Adult; **Taxon:** taxonID: https://www.gbif.org/species/7863197; scientificName: Odontomyia megacephala; **Location:** country: Egypt; locality: Cleopatra; decimalLatitude: 31.22021; decimalLongitude: 29.9348; **Identification:** identifiedBy: M. El-Hawagry & M. Hauser; dateIdentified: 2020-2021; **Event:** samplingProtocol: Not given; eventDate: 07-10-1921; **Record Level:** institutionCode: EFC
- **Type status:** Other material. **Occurrence:** recordedBy: Efflatoun; sex: 1 male; lifeStage: Adult; **Taxon:** taxonID: https://www.gbif.org/species/7863197; scientificName: Odontomyia megacephala; **Location:** country: Egypt; locality: Cleopatra; decimalLatitude: 31.22021; decimalLongitude: 29.9348; **Identification:** identifiedBy: M. El-Hawagry & M. Hauser; dateIdentified: 2020-2021; **Event:** samplingProtocol: Not given; eventDate: 07-22-1921; **Record Level:** institutionCode: EFC
- **Type status:** Other material. **Occurrence:** recordedBy: Efflatoun; sex: 1 male; lifeStage: Adult; **Taxon:** taxonID: https://www.gbif.org/species/7863197; scientificName: Odontomyia megacephala; **Location:** country: Egypt; locality: Cleopatra; decimalLatitude: 31.22021; decimalLongitude: 29.9348; **Identification:** identifiedBy: M. El-Hawagry & M. Hauser; dateIdentified: 2020-2021; **Event:** samplingProtocol: Not given; eventDate: 08-01-1921; **Record Level:** institutionCode: EFC
- **Type status:** Other material. **Occurrence:** recordedBy: Efflatoun; sex: 1 male; lifeStage: Adult; **Taxon:** taxonID: https://www.gbif.org/species/7863197; scientificName: Odontomyia megacephala; **Location:** country: Egypt; locality: Fayed; decimalLatitude: 30.32382; decimalLongitude: 32.3008; **Identification:** identifiedBy: M. El-Hawagry & M. Hauser; dateIdentified: 2020-2021; **Event:** samplingProtocol: Not given; eventDate: 09-24-1925; **Record Level:** institutionCode: EFC
- **Type status:** Other material. **Occurrence:** recordedBy: Efflatoun; sex: 1 female; lifeStage: Adult; **Taxon:** taxonID: https://www.gbif.org/species/7863197; scientificName: Odontomyia megacephala; **Location:** country: Egypt; locality: Fayed; decimalLatitude: 30.32382; decimalLongitude: 32.3008; **Identification:** identifiedBy: M. El-Hawagry & M. Hauser; dateIdentified: 2020-2021; **Event:** samplingProtocol: Not given; eventDate: 10-20-1925; **Record Level:** institutionCode: EFC
- **Type status:** Other material. **Occurrence:** recordedBy: Efflatoun; sex: 2 females; lifeStage: Adult; **Taxon:** taxonID: https://www.gbif.org/species/7863197; scientificName: Odontomyia megacephala; **Location:** country: Egypt; locality: Wadi El-Natroun; decimalLatitude: 30.3814; decimalLongitude: 30.3441; **Identification:** identifiedBy: M. El-Hawagry & M. Hauser; dateIdentified: 2020-2021; **Event:** samplingProtocol: Not given; eventDate: 08-06-1929; **Record Level:** institutionCode: EFC

##### Distribution

PA: Egypt. [Sources: Lindner (1936), Woodley (2001) and Badrawy (2006)]

**Figure 5.**
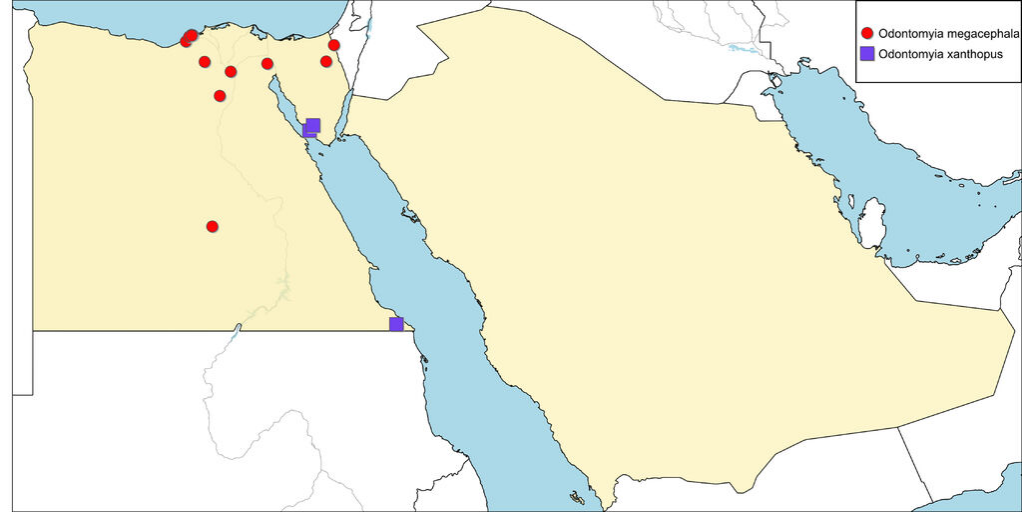
Distribution map of *Odontomyia megacephala* Olivier and *Odontomyia xanthopus* Bezzi.

**Local distribution and dates of collection** (Fig. 5): EGYPT: Coastal Strip: Alexandria, Cleopatra, Mariout, Max, Nuzha (May and August). Eastern Desert: Fayed (September). Fayoum: Fayoum City (September). Lower Nile Valley: El-Baragil (April). Sinai: Kosseimah, Moweileh (September). Western Desert: Kharga Oasis, Wadi El-Natroun (August to October). [Sources: original description (Olivier 1811), Badrawy (2006) and museum material]

#### Odontomyia xanthopus

Bezzi, 1906

2FC4B24A-43C0-510A-953E-49D8BC43F870

https://www.gbif.org/species/1581000

Odontomyia xanthopus Bezzi, 1906: 225. Type locality: Eritrea (Adi Ugri) [Adi Ugri belongs now to Eritrea not to Ethiopia as written in world catalogues].

##### Materials

- **Type status:** Other material. **Occurrence:** recordedBy: M.Tewfik; sex: 1 male, 3 females; lifeStage: Adult; **Taxon:** taxonID: https://www.gbif.org/species/1581000; scientificName: Odontomyia xanthopus; **Location:** country: Egypt; locality: Gebel Ela; decimalLatitude: 22.2008; decimalLongitude: 36.3331; **Identification:** identifiedBy: M. El-Hawagry & M. Hauser; dateIdentified: 2020-2021; **Event:** samplingProtocol: Not given; eventDate: Apr 1928; **Record Level:** institutionCode: EFC

##### Distribution

AF: Eritrea, Malawi, Oman, Yemen (this is the first time this species has been recorded from Oman and Yemen). PA: Egypt, Israel. [Sources: original description (Bezzi 1906), Woodley (2001) and collected material from Yemen and Oman]

**Local distribution and dates of collection** (Fig. 5): EGYPT: Gebel Elba: ? (April). Sinai: El-Tour, Wadi Hebran (May to July). [Source: Lindner (1930), Lindner (1974) and museum material]

#### Oplodontha

Rondani, 1863

8AAE7DD9-5859-5413-A56C-050816A7C31E

https://www.gbif.org/species/1577328

Oplodontha Rondani, 1863: 78. Type species: *Stratiomys viridula* Fabricius, by original designation.

#### Oplodontha minuta

Fabricius, 1794

7F02B74F-4E16-588A-8469-8893168BA54B

https://www.gbif.org/species/1577386

Stratiomys minuta Fabricius, 1794: 268. Type locality: India (Tranquebar).
Nemotelus pusillus Fabricius, 1794: 271. Type locality: India (Tranquebar).
Musca minutior Turton, 1801: 631. New name for Stratiomys minuta Fabricius, 1794.
Musca minor Turton, 1801: 655. New name for Nemotelus pusillus Fabricius, 1794.
Oxycera indica Brunetti, 1907: 119. Type locality: India (Uttar Pradesh: Bareilly).
Odontomyia incompleta Brunetti, 1907: 128. Nomen nudum.
Odontomyia ochracea Brunetti, 1907: 129. Type locality: India (Calcutta).
Odontomyia submutica Brunetti, 1907: 130. Type locality: India (Siliguri, Calcutta and Tollygunge).
Eulalia oasina Lindner, 1925: 150. Type locality: Egypt (Khârga Oasis and Dakhla Oasis).

##### Materials

- **Type status:** Other material. **Occurrence:** recordedBy: Efflatoun; lifeStage: Adult; **Taxon:** taxonID: https://www.gbif.org/species/1577386; scientificName: Oplodontha minuta; **Location:** country: Egypt; locality: Dakhla Oasis; decimalLatitude: 25.5; decimalLongitude: 29.1667; **Identification:** identifiedBy: M. El-Hawagry & M. Hauser; dateIdentified: 2020-2021; **Event:** samplingProtocol: Not given; eventDate: 05-13-2018; **Record Level:** institutionCode: ESEC
- **Type status:** Other material. **Occurrence:** recordedBy: Efflatoun; lifeStage: Adult; **Taxon:** taxonID: https://www.gbif.org/species/1577386; scientificName: Oplodontha minuta; **Location:** country: Egypt; locality: Ein Moussa; decimalLatitude: 29.8667; decimalLongitude: 32.65; **Identification:** identifiedBy: M. El-Hawagry & M. Hauser; dateIdentified: 2020-2021; **Event:** samplingProtocol: Not given; eventDate: 09-20-1924; **Record Level:** institutionCode: ESEC
- **Type status:** Other material. **Occurrence:** recordedBy: Storey; sex: 1 male; lifeStage: Adult; **Taxon:** taxonID: https://www.gbif.org/species/1577386; scientificName: as *Eulalia oasina*; **Location:** country: Egypt; locality: Kharga Oasis; decimalLatitude: 25.25; decimalLongitude: 30.5833; **Identification:** identifiedBy: M. El-Hawagry & M. Hauser; dateIdentified: 2020-2021; **Event:** samplingProtocol: Not given; eventDate: 05-10-1918; **Record Level:** institutionCode: EFC
- **Type status:** Other material. **Occurrence:** recordedBy: Efflatoun; sex: 1 female; lifeStage: Adult; **Taxon:** taxonID: https://www.gbif.org/species/1577386; scientificName: as *Eulalia oasina*; **Location:** country: Egypt; locality: Kharga Oasis; decimalLatitude: 25.25; decimalLongitude: 30.5833; **Identification:** identifiedBy: M. El-Hawagry & M. Hauser; dateIdentified: 2020-2021; **Event:** samplingProtocol: Not given; eventDate: 06-12-1918; **Record Level:** institutionCode: EFC

##### Distribution

AF: Socotra Island, United Arab Emirates, Yemen. OR: India, Sri Lanka. PA: Afghanistan, Egypt, Israel. [Sources: original description of *O. oasina* (Lindner 1925), Woodley (2001) and Tkoč and Rozkošný (2014)]

**Figure 6.**
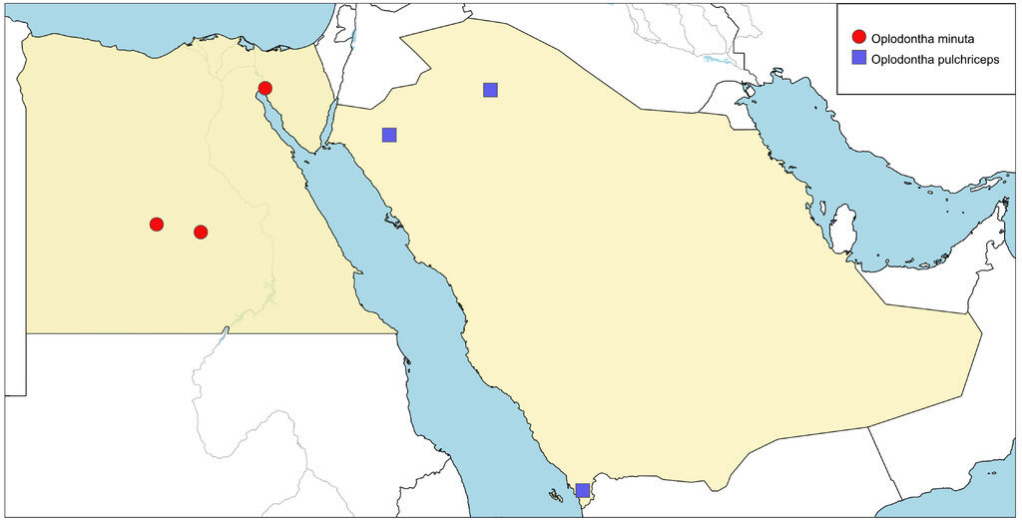
Distribution map of *Oplodontha minuta* Fabricius and *Oplodontha pulchriceps* Loew.

**Local distribution and dates of collection** (Fig. 6): EGYPT: Eastern Desert: Ein Moussa (September). Western Desert: Dakhla Oasis, Kharga Oasis (May and June). [Sources: Lindner (1925), Lindner (1930) and museum material]

#### Oplodontha pulchriceps

Loew, 1858

3A60294B-A809-53E8-9EB9-788A7EF5F301

https://www.gbif.org/species/1577339

Odontomyia pulchriceps Loew, 1858: 335. Type locality: South Africa (Cape of Good Hope).
Odontomyia pulchriceps Loew, 1860: 80. Type locality: South Africa (Cape of Good Hope). Preoccupied, primary homonym of *Odontomyia pulchriceps* Loew, 1858.
Hoplodonta madagascariensis Lindner, 1936: 42. Type locality: Madagascar (Bekily).

##### Materials

- **Type status:** Other material. **Occurrence:** recordedBy: Aldhafer H.M. et al.; sex: 1 male; lifeStage: Adult; **Taxon:** taxonID: https://www.gbif.org/species/1577339; scientificName: Oplodontha pulchriceps; **Location:** country: Saudi Arabia; locality: Jazan; decimalLatitude: 16.9595; decimalLongitude: 42.8348; **Identification:** identifiedBy: M. El-Hawagry & M. Hauser; dateIdentified: 2020-2021; **Event:** samplingProtocol: Malaise trap; eventDate: 05-11-2018; **Record Level:** institutionCode: KSMA
- **Type status:** Other material. **Occurrence:** recordedBy: Aldhafer H.M. et al.; sex: 1 male; lifeStage: Adult; **Taxon:** taxonID: https://www.gbif.org/species/1577339; scientificName: Oplodontha pulchriceps; **Location:** country: Saudi Arabia; locality: Dawmat Al-Jandal; decimalLatitude: 29.809552; decimalLongitude: 39.8749; **Identification:** identifiedBy: M. El-Hawagry & M. Hauser; dateIdentified: 2020-2021; **Event:** samplingProtocol: Malaise trap; eventDate: 05-26-2018; **Record Level:** institutionCode: KSMA
- **Type status:** Other material. **Occurrence:** recordedBy: Aldhafer H.M. et al.; sex: 1 female; lifeStage: Adult; **Taxon:** taxonID: https://www.gbif.org/species/1577339; scientificName: Oplodontha pulchriceps; **Location:** country: Saudi Arabia; locality: Hassan Ameen farm; decimalLatitude: 28.36661; decimalLongitude: 36.6297; **Identification:** identifiedBy: M. El-Hawagry & M. Hauser; dateIdentified: 2020-2021; **Event:** samplingProtocol: Sweeping; eventDate: 05-27-2018; **Record Level:** institutionCode: KSMA

##### Distribution

AF: Democratic Republic of Congo, Lesotho, Madagascar, Mozambique, Saudi Arabia [as “south western part"], South Africa, United Arab Emirates. PA: Israel, Saudi Arabia [Jawf and Tabouk]. [Sources: Woodley (2001), Hauser (2008) and collected material]

**Local distribution and dates of collection** (Fig. 6): SAUDI ARABIA: Al-Jawf: Dawmat Al-Jandal (May). Jazan: Jazan (May). Tabouk: Hassan Ameen farm (May). [Source: collected material]

##### Notes

**Figure 7.**
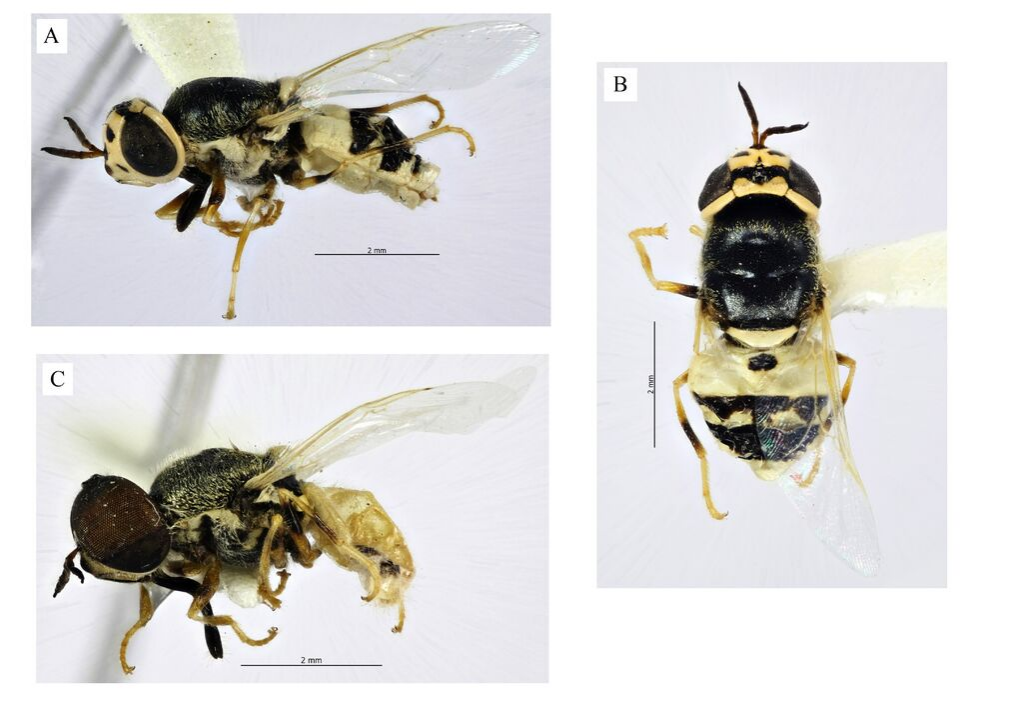
*Oplodontha pulchriceps* Loew: **A.** female habitus, lateral; **B.** same, dorsal; **C.** male habitus, lateral.

This species (Fig. 7) and the genus *Oplodontha* are recorded herein for the first time from Saudi Arabia. It looks similar to *O. minuta* Fabricius; however, this case requires more clarification in a future study.

#### Stratiomys

Geoffroy, 1762

8AB342A4-CA7D-5E19-B448-513C3B81C41C

https://www.gbif.org/species/1501902

Stratiomys Geoffroy, 1762: 449, 475. Type species: *Musca chamaeleon* Linnaeus, by designation of I.C.Z.N. (1957).

#### Stratiomys cenisia

Meigen, 1822

FAACD0CB-FF58-5ABC-A724-35427CE6C111

https://www.gbif.org/species/1577708

Stratiomys cenisia Meigen, 1822: 136. Type locality: France (Mont Cenis).
Stratiomys flaviventris Loew, 1846: 464. Type locality: Italy (Sicily: Siracusa).
Stratiomyia ahngeri Pleske, 1901: 364. Type locality: “Transcaspian Region” [probably = Turkmenistan].
Stratiomyia cypria Pleske, 1902: 413. Type locality: Cyprus (Lárnax).
Stratiomyia kervillei Villeneuve, 1911: 4. Type locality: Syria (near “lac de Homs”).
Stratiomys hispanica ssp. planes James, 1941: 18. Type locality: Iran (Curum, 100 km from Bouchir).

##### Distribution

PA: Algeria, Armenia, Austria, Bulgaria, Cyprus, Czech Republic, Egypt, France, Germany, Hungary, Iran, Israel, Italy, Kazakhstan, Morocco, Poland, Romania, Russia, Slovakia, Spain, Syria, Tunisia, Turkey, Turkmenistan, Ukraine, former Yugoslavia. [Sources: Woodley (2001) and Yimlahi et al. (2017)]

**Local distribution and dates of collection**: Unknown.

##### Notes

This species was listed by Woodley (2001), Badrawy (2006) and Yimlahi et al. (2017) as recorded from Egypt, but no specimens have been collected or preserved in Egyptian museums to verify these records.

#### Stratiomys deserticolor

Lindner, 1930

F948E65B-43AB-5508-B4DB-AF868AB059B2

https://www.gbif.org/species/4293811

Stratiomyia segnis form deserticolor Lindner, 1930: 27. Type locality: Egypt (Siwa Oasis).

##### Materials

- **Type status:** Other material. **Occurrence:** recordedBy: Efflatoun; sex: 1 male; lifeStage: Adult; **Taxon:** taxonID: https://www.gbif.org/species/4293811; scientificName: Stratiomys deserticolor; **Location:** country: Egypt; locality: Kharga Oasis; decimalLatitude: 25.2500; decimalLongitude: 30.5833; **Identification:** identifiedBy: M. El-Hawagry & M. Hauser; dateIdentified: 2020-2021; **Event:** samplingProtocol: Not given; eventDate: 08-12-1926; **Record Level:** institutionCode: EFC
- **Type status:** Other material. **Occurrence:** recordedBy: Efflatoun; sex: 1 female; lifeStage: Adult; **Taxon:** taxonID: https://www.gbif.org/species/4293811; scientificName: Stratiomys deserticolor; **Location:** country: Egypt; locality: Mariout; decimalLatitude: 31.0172; decimalLongitude: 29.76; **Identification:** identifiedBy: M. El-Hawagry & M. Hauser; dateIdentified: 2020-2021; **Event:** samplingProtocol: Not given; eventDate: Jul 1934; **Record Level:** institutionCode: EFC

##### Distribution

PA: Egypt, Saudi Arabia. [Sources: original description (Lindner 1930), Abu-Zoherah et al. (1993) and Woodley (2001)]

**Figure 8.**
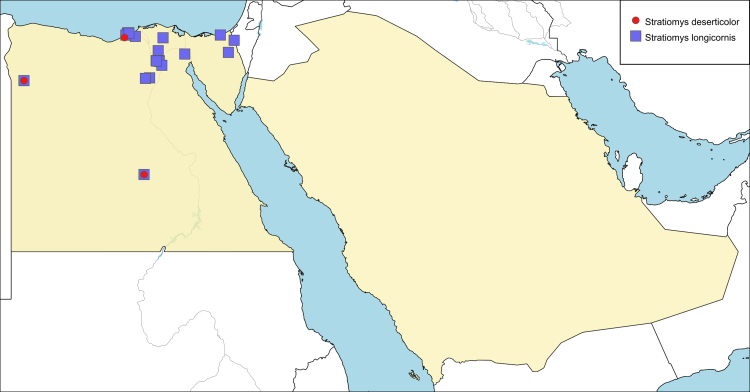
Distribution map of *Stratiomys deserticolor* Lindner and *Stratiomys longicornis* (Scopoli).

**Local distribution and dates of collection** (Fig. 8): EGYPT: Coastal Strip: Mariout (July). Western Desert: Kharga Oasis, Siwa Oasis (February to August) [Sources: Original description (Lindner 1930) and museum material]. SAUDI ARABIA: localities and dates unknown. [Sources: Abu-Zoherah et al. (1993)]

#### Stratiomys longicornis

(Scopoli, 1763)

4129030C-6E96-52DF-A33B-DD25BBBD46C9

https://www.gbif.org/species/1577660

Hirtea longicornis Scopoli, 1763: 367. Type locality: “Carniola” [= Slovenija?].
Musca tenebricus Harris, 1778: 45. Type locality: England.
Stratiomys strigata Fabricius, 1781: 417. Type locality: Italy.
Stratiomys tomentosa Schrank, 1803: 94. Type locality: Germany (Ingolstadt).
Stratiomys villosa Meigen, 1804: 124. Type locality: Europe.
Stratiomys nubeculosa Meigen, 1804: 125. Type locality: Europe.
Stratiomys thoracica Fabricius, 1805: 79. Type locality: France.
Stratiomys hirtuosa Meigen, 1830: 347. Type locality: Europe.
Stratiomys anubis Wiedemann, 1830: 60. Type locality: Egypt.
Stratiomyia flavifrons Macquart, 1838: 179. Type locality: “Mesopotamie” [= Iraq].
Stratiomys strigata var. pallida Loew, 1840: 25. Type locality: Poland (Poznañ).
Stratiomys strigata var. pallida Loew, 1840: 557. Type locality: Poland (Poznañ). Preoccupied, primary homonym of Stratiomys strigata var. pallida Loew, 1840.
Stratiomys lambessiana Bigot, 1879: 212. Type locality: Algeria (Lambessa).
Stratiomys flavolimbata Costa, 1893: 21. Type locality: Tunisia.
Stratiomyia pleskei Wagner, 1903: 108. Type locality: Uzbekistan (Fergana).
Stratiomyia segnis Becker, 1906: 8. Type locality: Tunisia (vicinity of Tunis).
Hirtea efflatouni Lindner, 1925: 148. Type locality: Egypt (Giza).
Stratiomyia (Hirtea) surcoufi Séguy, 1930: 63. Type locality: Algeria (Touggourt).
Hirtea surcoufi Séguy, 1932: 125. Type locality: Algeria (Touggourt). Preoccupied, secondary homonym of *Stratiomyia surcoufi* Séguy, 1930.
Stratiomyia longicornis ssp. palaestinensis Lindner, 1937: 64. Type locality: Israel (Upper-Galilee, Kfar-Giladi).
Stratiomyia (Hirtea) longicornis ssp. flavoscutellata Lindner, 1940: 24. Type locality: China (Shanxi Province: “Ta-tong-fou”). Preoccupied, primary homonym of *Stratiomyia flavoscutellata* Wulp, 1885.

##### Materials

- **Type status:** Other material. **Occurrence:** recordedBy: Efflatoun; lifeStage: Adult; **Taxon:** taxonID: https://www.gbif.org/species/1577660; scientificName: Stratiomys longicornis; **Location:** country: Egypt; locality: Siala; decimalLatitude: 31.20849; decimalLongitude: 29.8805; **Identification:** identifiedBy: M. El-Hawagry & M. Hauser; dateIdentified: 2020-2021; **Event:** samplingProtocol: Not given; eventDate: Nov 1913; **Record Level:** institutionCode: ESEC
- **Type status:** Other material. **Occurrence:** recordedBy: Efflatoun; lifeStage: Adult; **Taxon:** taxonID: https://www.gbif.org/species/1577660; scientificName: Stratiomys longicornis; **Location:** country: Egypt; locality: Fayed; decimalLatitude: 30.32382; decimalLongitude: 32.3008; **Identification:** identifiedBy: M. El-Hawagry & M. Hauser; dateIdentified: 2020-2021; **Event:** samplingProtocol: Not given; eventDate: Oct 1924; **Record Level:** institutionCode: ESEC
- **Type status:** Other material. **Occurrence:** recordedBy: Efflatoun; lifeStage: Adult; **Taxon:** taxonID: https://www.gbif.org/species/1577660; scientificName: Stratiomys longicornis; **Location:** country: Egypt; locality: Marg; decimalLatitude: 31.0667; decimalLongitude: 30.2167; **Identification:** identifiedBy: M. El-Hawagry & M. Hauser; dateIdentified: 2020-2021; **Event:** samplingProtocol: Not given; eventDate: 03-24-1918; **Record Level:** institutionCode: ESEC
- **Type status:** Other material. **Occurrence:** recordedBy: Efflatoun; lifeStage: Adult; **Taxon:** taxonID: https://www.gbif.org/species/1577660; scientificName: Stratiomys longicornis; **Location:** country: Egypt; locality: Kosseima; decimalLatitude: 30.90307; decimalLongitude: 34.3836; **Identification:** identifiedBy: M. El-Hawagry & M. Hauser; dateIdentified: 2020-2021; **Event:** samplingProtocol: Not given; eventDate: Aug 1924; **Record Level:** institutionCode: ESEC
- **Type status:** Other material. **Occurrence:** recordedBy: Efflatoun; lifeStage: Adult; **Taxon:** taxonID: https://www.gbif.org/species/1577660; scientificName: Stratiomys longicornis; **Location:** country: Egypt; locality: Moweileh; decimalLatitude: 30.3924; decimalLongitude: 34.1412; **Identification:** identifiedBy: M. El-Hawagry & M. Hauser; dateIdentified: 2020-2021; **Event:** samplingProtocol: Not given; eventDate: 08-21-1924; **Record Level:** institutionCode: ESEC
- **Type status:** Other material. **Occurrence:** recordedBy: Efflatoun; lifeStage: Adult; **Taxon:** taxonID: https://www.gbif.org/species/1577660; scientificName: Stratiomys longicornis; **Location:** country: Egypt; locality: Helwan; decimalLatitude: 29.8500; decimalLongitude: 31.3333; **Identification:** identifiedBy: M. El-Hawagry & M. Hauser; dateIdentified: 2020-2021; **Event:** samplingProtocol: Not given; eventDate: 11-25-1913; **Record Level:** institutionCode: ESEC
- **Type status:** Other material. **Occurrence:** recordedBy: Efflatoun; lifeStage: Adult; **Taxon:** taxonID: https://www.gbif.org/species/1577660; scientificName: Stratiomys longicornis; **Location:** country: Egypt; locality: Benha; decimalLatitude: 30.46572; decimalLongitude: 31.18121; **Identification:** identifiedBy: M. El-Hawagry & M. Hauser; dateIdentified: 2020-2021; **Event:** samplingProtocol: Not given; eventDate: 05-09-1918; **Record Level:** institutionCode: ESEC
- **Type status:** Other material. **Occurrence:** recordedBy: Efflatoun; lifeStage: Adult; **Taxon:** taxonID: https://www.gbif.org/species/1577660; scientificName: Stratiomys longicornis; **Location:** country: Egypt; locality: Gezeira; decimalLatitude: 30.04596; decimalLongitude: 31.22435; **Identification:** identifiedBy: M. El-Hawagry & M. Hauser; dateIdentified: 2020-2021; **Event:** samplingProtocol: Not given; eventDate: 09-21-1917; **Record Level:** institutionCode: ESEC
- **Type status:** Other material. **Occurrence:** recordedBy: Efflatoun; lifeStage: Adult; **Taxon:** taxonID: https://www.gbif.org/species/1577660; scientificName: Stratiomys longicornis; **Location:** country: Egypt; locality: Kharga Oasis; decimalLatitude: 25.25; decimalLongitude: 30.5833; **Identification:** identifiedBy: M. El-Hawagry & M. Hauser; dateIdentified: 2020-2021; **Event:** samplingProtocol: Not given; eventDate: 01-03-1914; **Record Level:** institutionCode: ESEC

##### Distribution

PA: Afghanistan, Albania, Algeria, Armenia, Austria, Azerbaijan, Belgium, Bulgaria, China, Cyprus, Czech Republic, Denmark, Egypt, England, France, Germany, Greece, Hungary, Iran, Israel, Italy including Sardinia, Korea, Lithuania, Malta, Mongolia, Morocco, Netherlands, Poland, Portugal, Romania, Russia, Scotland, Slovakia, Slovenia, Spain, Sweden, Switzerland, Tunisia, Turkey, former Yugoslavia. [Sources: Woodley (2001), Mason et al. (2009) and Yimlahi et al. (2017)]

**Local distribution and dates of collection** (Fig. 8): EGYPT: Coastal Strip: El-Siala, Cleopatra, Dekhela, Nuzha (April to November). Eastern Desert: Fayed (October). Fayoum: Fayoum City, Nazla, Sanhur (April, May and September). Lower Nile Valley and Delta: Abu-Rawash, Benha, Faraskour, Gezeira, Giza, Helwan, Kerdassa, Marg, Sandoub, Shubra (March to November). Sinai: El-Arish, Kosseimah, Moweileh (April and August). Western Desert: Kharga Oasis, Siwa Oasis (January and May). [Sources: Lindner (1925) and museum material]

#### Stratiomys singularior

(Harris, 1776)

331107BE-392C-566A-A150-01AE52A595BC

https://www.gbif.org/species/4293822

Musca singularius Harris, 1778: 45. Type locality: England.
Stratiomys furcata Fabricius, 1794: 264. Type locality: Germany.
Stratiomys panthaleon Fallén, 1817: 7. Type locality: Sweden.
Stratiomys riparia Meigen, 1822: 138. Type locality: Europe.
Stratiomys paludosa Siebke, 1863: 149. Type locality: Norway (Dovre Mountains: Jerkin). Preoccupied, primary homonym of *Stratiomys paludosa* Schummel in Gravenhorst, 1837.

##### Materials

- **Type status:** Other material. **Occurrence:** sex: 1 male; lifeStage: Adult; **Taxon:** taxonID: https://www.gbif.org/species/4293822; scientificName: Stratiomys singularior; **Location:** country: Egypt; locality: Fayoum; decimalLatitude: 29.32061; decimalLongitude: 30.818; **Identification:** identifiedBy: M. El-Hawagry & M. Hauser; dateIdentified: 2020-2021; **Event:** samplingProtocol: Not given; eventDate: 03-01-1947; **Record Level:** institutionCode: EFC
- **Type status:** Other material. **Occurrence:** sex: 2 males; lifeStage: Adult; **Taxon:** taxonID: https://www.gbif.org/species/4293822; scientificName: Stratiomys singularior; **Location:** country: Egypt; locality: Girza; decimalLatitude: 29.49968; decimalLongitude: 31.0738; **Identification:** identifiedBy: M. El-Hawagry & M. Hauser; dateIdentified: 2020-2021; **Event:** samplingProtocol: Not given; eventDate: 04-09-1950; **Record Level:** institutionCode: EFC
- **Type status:** Other material. **Occurrence:** recordedBy: Efflatoun; sex: 1 male; lifeStage: Adult; **Taxon:** taxonID: https://www.gbif.org/species/4293822; scientificName: Stratiomys singularior; **Location:** country: Egypt; locality: Abu-Zaabal; decimalLatitude: 30.24098; decimalLongitude: 31.35211; **Identification:** identifiedBy: M. El-Hawagry & M. Hauser; dateIdentified: 2020-2021; **Event:** samplingProtocol: Not given; eventDate: 03-19-1950; **Record Level:** institutionCode: EFC

##### Distribution

PA: Armenia, Austria, Belgium, Bulgaria, China, Czech Republic, Denmark, Egypt, England, Estonia, Finland, France, Germany, Hungary, Iran, Ireland, Italy, Kazakhstan, Lithuania, Mongolia, Netherlands, Norway, Poland, Romania, Russia, Slovakia, Spain, Sweden, Switzerland, Ukraine, former Yugoslavia. [Sources: Woodley (2001) and Badrawy (2006)]

**Figure 9.**
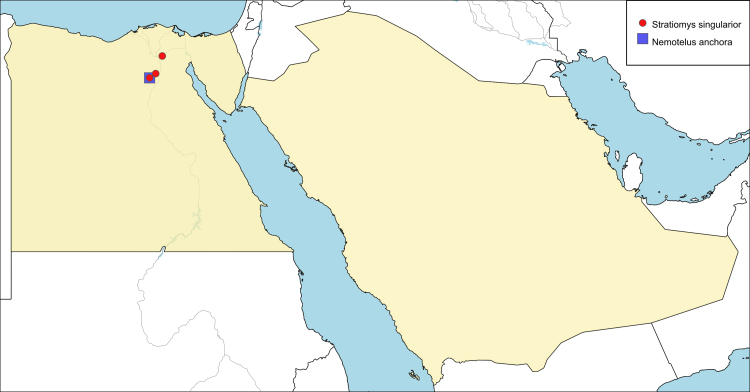
Distribution map of *Stratiomys singularior* (Harris) and *Nemotelus anchora* Loew.

**Local distribution and dates of collection** (Fig. 9): EGYPT: Fayoum: Fayoum City, Girza (March and April). Lower Nile Valley & Delta: Abu-Zaabal (March). [Source: Badrawy (2006) and museum material]

#### Nemotelinae

2ABD0C30-4A87-5082-B24B-69C3CF321C1A

#### Nemotelus

Geoffroy, 1762

49710B4B-7BC2-5B6B-A8E6-E4102126E652

https://www.gbif.org/species/1501964

Nemotelus Geoffroy, 1762: 450, 542. Type species: *Musca pantherina* Linnaeus, by designation of I.C.Z.N. (1957).

#### Nemotelus

Geoffroy, 1762

059ED454-0BEF-53E2-A1DF-D786D1F51441

#### Nemotelus (Nemotelus) anchora

Loew, 1846

4D1316EF-9C6F-5EC9-BE6E-C597F3125FD4

https://www.gbif.org/species/1578822

Nemotelus anchora Loew, 1846: 429. Type locality: Italy (Sicily: Siracusa).
Nemotelus siculus Jaennicke, 1866: 224. Type locality: Italy (Sicily).
Nemotelus persicus Pleske in Lindner, 1937: 137. Type locality: Iran (Irak-Adzhemi: Buyun Village).

##### Materials

- **Type status:** Other material. **Occurrence:** recordedBy: Efflatoun; sex: 32 males, 5 females; lifeStage: Adult; **Taxon:** taxonID: https://www.gbif.org/species/1578822; scientificName: Nemotelus anchora; **Location:** country: Egypt; locality: Fayoum; decimalLatitude: 29.32061; decimalLongitude: 30.818; **Identification:** identifiedBy: M. El-Hawagry & M. Hauser; dateIdentified: 2020-2021; **Event:** samplingProtocol: Not given; eventDate: 03-01-1947; **Record Level:** institutionCode: EFC

##### Distribution

PA: Algeria, Egypt, Iran, Israel, Italy (including Sicily and Sardinia), Malta, Russia, Tunisia. [Sources: Woodley (2001), Mason et al. (2009) and Mohammad et al. (2009)]

**Local distribution and dates of collection** (Fig. 9): EGYPT: Fayoum: ? (March). [Source: Mohammad et al. (2009)]

#### Nemotelus (Nemotelus) candidus

Becker, 1906

D2323DD6-1BD1-51F5-B458-C7959CF4432A

https://www.gbif.org/species/1578675

Nemotelus candidus Becker, 1906: 4. Type locality: Algeria (Biskra: Hammam-Salahin).

##### Materials

- **Type status:** Other material. **Occurrence:** recordedBy: Efflatoun; sex: 4 males, 1 female; lifeStage: Adult; **Taxon:** taxonID: https://www.gbif.org/species/1578675; scientificName: Nemotelus candidus; **Location:** country: Egypt; locality: Wadi El-Natroun; decimalLatitude: 30.3814; decimalLongitude: 30.3441; **Identification:** identifiedBy: M. El-Hawagry & M. Hauser; dateIdentified: 2020-2021; **Event:** samplingProtocol: Not given; eventDate: 08-06-1929; **Record Level:** institutionCode: EFC

##### Distribution

PA: Algeria, Egypt. [Sources: Woodley (2001) and Mohammad et al. (2009)]

**Figure 10.**
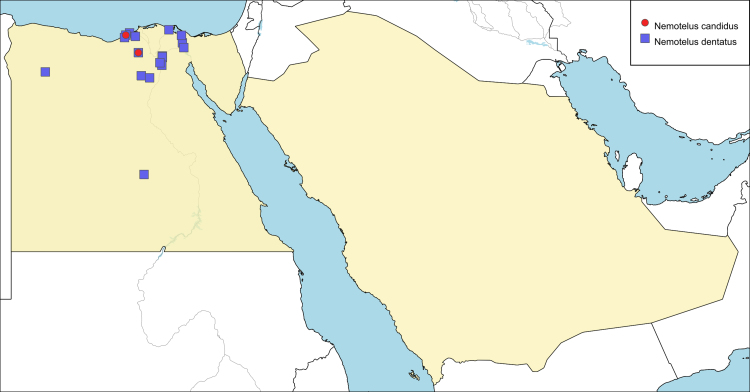
Distribution map of *Nemotelus candidus* Becker and *Nemotelus dentatus* Becker.

**Local distribution and dates of collection** (Fig. 10): EGYPT: Coastal Strip: Dekhela (May and September). Western Desert: Wadi El-Natroun (August). [Source: Mohammad et al. (2009)]

#### Nemotelus (Nemotelus) dentatus

Becker, 1902

CA086D1F-AD03-5D5E-8BB4-6CD0A066026E

https://www.gbif.org/species/1578829

Nemotelus dentatus Becker, 1902: 7. Type localities: Egypt (Birket Qaroun, Damietta and Alexandria).

##### Materials

- **Type status:** Other material. **Occurrence:** recordedBy: Efflatoun; sex: 1 female; lifeStage: Adult; **Taxon:** taxonID: https://www.gbif.org/species/1578829; scientificName: Nemotelus dentatus; **Location:** country: Egypt; locality: Maadi; decimalLatitude: 29.95772; decimalLongitude: 31.2505; **Identification:** identifiedBy: M. El-Hawagry & M. Hauser; dateIdentified: 2020-2021; **Event:** samplingProtocol: Not given; eventDate: 06-05-1916; **Record Level:** institutionCode: ESEC
- **Type status:** Other material. **Occurrence:** recordedBy: Efflatoun; sex: 1 female; lifeStage: Adult; **Taxon:** taxonID: https://www.gbif.org/species/1578829; scientificName: Nemotelus dentatus; **Location:** country: Egypt; locality: Helwan; decimalLatitude: 29.8500; decimalLongitude: 31.3333; **Identification:** identifiedBy: M. El-Hawagry & M. Hauser; dateIdentified: 2020-2021; **Event:** samplingProtocol: Not given; eventDate: 02-11-1924; **Record Level:** institutionCode: ESEC
- **Type status:** Other material. **Occurrence:** recordedBy: Efflatoun; sex: 1 male; lifeStage: Adult; **Taxon:** taxonID: https://www.gbif.org/species/1578829; scientificName: Nemotelus dentatus; **Location:** country: Egypt; locality: Helwan; decimalLatitude: 29.8500; decimalLongitude: 31.3333; **Identification:** identifiedBy: M. El-Hawagry & M. Hauser; dateIdentified: 2020-2021; **Event:** samplingProtocol: Not given; eventDate: 02-12-1924; **Record Level:** institutionCode: ESEC
- **Type status:** Other material. **Occurrence:** recordedBy: Efflatoun; sex: 2 males, 2 females; lifeStage: Adult; **Taxon:** taxonID: https://www.gbif.org/species/1578829; scientificName: Nemotelus dentatus; **Location:** country: Egypt; locality: Fayoum; decimalLatitude: 29.32061; decimalLongitude: 30.818; **Identification:** identifiedBy: M. El-Hawagry & M. Hauser; dateIdentified: 2020-2021; **Event:** samplingProtocol: Not given; eventDate: 03-01-1947; **Record Level:** institutionCode: EFC
- **Type status:** Other material. **Occurrence:** recordedBy: Efflatoun; sex: 1 female; lifeStage: Adult; **Taxon:** taxonID: https://www.gbif.org/species/1578829; scientificName: Nemotelus dentatus; **Location:** country: Egypt; locality: Marg; decimalLatitude: 31.0667; decimalLongitude: 30.2167; **Identification:** identifiedBy: M. El-Hawagry & M. Hauser; dateIdentified: 2020-2021; **Event:** samplingProtocol: Not given; eventDate: 04-01-1923; **Record Level:** institutionCode: EFC
- **Type status:** Other material. **Occurrence:** recordedBy: Efflatoun; sex: 1 female; lifeStage: Adult; **Taxon:** taxonID: https://www.gbif.org/species/1578829; scientificName: Nemotelus dentatus; **Location:** country: Egypt; locality: Helwan; decimalLatitude: 29.8500; decimalLongitude: 31.3333; **Identification:** identifiedBy: M. El-Hawagry & M. Hauser; dateIdentified: 2020-2021; **Event:** samplingProtocol: Not given; eventDate: 04-02-1939; **Record Level:** institutionCode: EFC
- **Type status:** Other material. **Occurrence:** recordedBy: Efflatoun; sex: 2 males; lifeStage: Adult; **Taxon:** taxonID: https://www.gbif.org/species/1578829; scientificName: Nemotelus dentatus; **Location:** country: Egypt; locality: Kharga Oasis; decimalLatitude: 25.2500; decimalLongitude: 30.5833; **Identification:** identifiedBy: M. El-Hawagry & M. Hauser; dateIdentified: 2020-2021; **Event:** samplingProtocol: Not given; eventDate: 03-13-1932; **Record Level:** institutionCode: EFC
- **Type status:** Other material. **Occurrence:** recordedBy: Efflatoun; sex: 2 males; lifeStage: Adult; **Taxon:** taxonID: https://www.gbif.org/species/1578829; scientificName: Nemotelus dentatus; **Location:** country: Egypt; locality: Mirsa Matrouh; decimalLatitude: 29.5696; decimalLongitude: 26.4194; **Identification:** identifiedBy: M. El-Hawagry & M. Hauser; dateIdentified: 2020-2021; **Event:** samplingProtocol: Not given; eventDate: 05-08-1935; **Record Level:** institutionCode: EFC
- **Type status:** Other material. **Occurrence:** recordedBy: Tewfik; sex: 2 males, 2 females; lifeStage: Adult; **Taxon:** taxonID: https://www.gbif.org/species/1578829; scientificName: Nemotelus dentatus; **Location:** country: Egypt; locality: Ismaila; decimalLatitude: 30.32382; decimalLongitude: 32.3008; **Identification:** identifiedBy: M. El-Hawagry & M. Hauser; dateIdentified: 2020-2021; **Event:** samplingProtocol: Not given; eventDate: 04-07-1924; **Record Level:** institutionCode: EFC
- **Type status:** Other material. **Occurrence:** recordedBy: Efflatoun; sex: 2 females; lifeStage: Adult; **Taxon:** taxonID: https://www.gbif.org/species/1578829; scientificName: Nemotelus dentatus; **Location:** country: Egypt; locality: Ismaila; decimalLatitude: 30.32382; decimalLongitude: 32.3008; **Identification:** identifiedBy: M. El-Hawagry & M. Hauser; dateIdentified: 2020-2021; **Event:** samplingProtocol: Not given; eventDate: 10-14-1926; **Record Level:** institutionCode: EFC
- **Type status:** Other material. **Occurrence:** recordedBy: Efflatoun; sex: 2 males; lifeStage: Adult; **Taxon:** taxonID: https://www.gbif.org/species/1578829; scientificName: Nemotelus dentatus; **Location:** country: Egypt; locality: Ismaila; decimalLatitude: 30.32382; decimalLongitude: 32.3008; **Identification:** identifiedBy: M. El-Hawagry & M. Hauser; dateIdentified: 2020-2021; **Event:** samplingProtocol: Not given; eventDate: 10-14-1926; **Record Level:** institutionCode: EFC
- **Type status:** Other material. **Occurrence:** recordedBy: Efflatoun; sex: 1 male; lifeStage: Adult; **Taxon:** taxonID: https://www.gbif.org/species/1578829; scientificName: Nemotelus dentatus; **Location:** country: Egypt; locality: Dekhela; decimalLatitude: 31.12098; decimalLongitude: 29.8157; **Identification:** identifiedBy: M. El-Hawagry & M. Hauser; dateIdentified: 2020-2021; **Event:** samplingProtocol: Not given; eventDate: May-April 1923; **Record Level:** institutionCode: EFC
- **Type status:** Other material. **Occurrence:** recordedBy: Efflatoun; sex: 1 female; lifeStage: Adult; **Taxon:** taxonID: https://www.gbif.org/species/1578829; scientificName: Nemotelus dentatus; **Location:** country: Egypt; locality: Abu-Zaabal; decimalLatitude: 30.24098; decimalLongitude: 31.3521; **Identification:** identifiedBy: M. El-Hawagry & M. Hauser; dateIdentified: 2020-2021; **Event:** samplingProtocol: Not given; eventDate: 03-19-1950; **Record Level:** institutionCode: EFC

##### Distribution

PA: Egypt. [Source: Woodley (2001)]

**Local distribution and dates of collection** (Fig. 10): EGYPT: Coastal Strip: Alexandria, Dekhela, Mariout, Mersa Matrouh (March to May). Eastern Desert: Ismailia, El-Kantara (April, May & October). Fayoum: Birket Qaroun, El-Athar [(April & May). Lower Nile Valley and Delta: Abu-Zaabal, Damietta, El-Alag to Marg, El-Gabal El-Asfar, Ezbet El-Nakhl, Helwan, Maadi (February to April & November). Western Desert: Kharga Oasis, Wadi El-Natroun (March). [Sources: original description Becker (1902), Lindner (1925), Mohammad et al. (2009) and museum material]

#### Nemotelus (Nemotelus) marinus

Becker, 1902

E9E34996-28CE-5C91-9FA0-B7D217AF6AF6

https://www.gbif.org/species/1578792

Nemotelus marinus Becker, 1902: 9. Type locality: Egypt (Suez, at the seashore).

##### Materials

- **Type status:** Other material. **Occurrence:** recordedBy: Efflatoun; sex: 1 male; lifeStage: Adult; **Taxon:** taxonID: https://www.gbif.org/species/1578792; scientificName: Nemotelus marinus; **Location:** country: Egypt; locality: Fayed; decimalLatitude: 30.32382; decimalLongitude: 32.3008; **Identification:** identifiedBy: M. El-Hawagry & M. Hauser; dateIdentified: 2020-2021; **Event:** samplingProtocol: Not given; eventDate: 09-24-1925; **Record Level:** institutionCode: EFC
- **Type status:** Other material. **Occurrence:** recordedBy: Efflatoun; sex: 1 female; lifeStage: Adult; **Taxon:** taxonID: https://www.gbif.org/species/1578792; scientificName: Nemotelus marinus; **Location:** country: Egypt; locality: Wadi Hoff; decimalLatitude: 29.8821; decimalLongitude: 31.311; **Identification:** identifiedBy: M. El-Hawagry & M. Hauser; dateIdentified: 2020-2021; **Event:** samplingProtocol: Not given; eventDate: 08-09-1927; **Record Level:** institutionCode: EFC
- **Type status:** Other material. **Occurrence:** recordedBy: Efflatoun; sex: 1 female; lifeStage: Adult; **Taxon:** taxonID: https://www.gbif.org/species/1578792; scientificName: Nemotelus marinus; **Location:** country: Egypt; locality: Wadi El-Natroun; decimalLatitude: 30.3814; decimalLongitude: 30.3441; **Identification:** identifiedBy: M. El-Hawagry & M. Hauser; dateIdentified: 2020-2021; **Event:** samplingProtocol: Not given; eventDate: 08-06-1929; **Record Level:** institutionCode: EFC

##### Distribution

PA: Egypt. [Source: original description Becker (1902) and Woodley (2001)]

**Figure 11.**
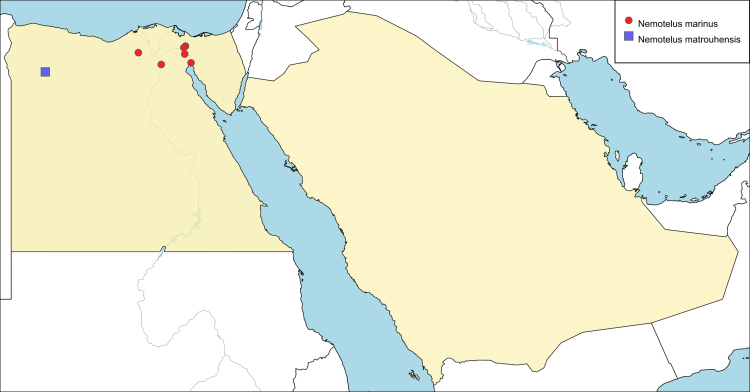
Distribution map of *Nemotelus marinus* Becker and *Nemotelus matrouhensis* Mohammad et al.

**Local distribution and dates of collection** (Fig. 11): EGYPT: Eastern Desert: El-Ferdan, Fayed, Ismailia, Suez, Wadi Hoff (April to October). Western Desert: Wadi El Natroun (August). [Sources: original description (Becker 1902), Mohammad et al. (2009) and museum material]

#### Nemotelus (Nemotelus) matrouhensis

Mohammad et al., 2009

2097C6B9-034B-5F2F-B299-255920000E98

Nemotelus matrouhensis Mohammad, Fadl, Gadalla & Badrawy, 2009: 103. Type locality: Egypt (Mersa Matrouh).

##### Materials

- **Type status:** Lectotype. **Occurrence:** recordedBy: H.C.E & M.T.; sex: male; lifeStage: Adult; **Taxon:** scientificName: Nemotelus matrouhensis; **Location:** country: Egypt; locality: Mersa Matrouh; decimalLatitude: 29.5696; decimalLongitude: 26.4194; **Identification:** identifiedBy: M. El-Hawagry & M. Hauser; dateIdentified: 2020-2021; **Event:** samplingProtocol: Not given; eventDate: July & Aug 1931; **Record Level:** institutionCode: EFC

##### Distribution

PA: Egypt. [Source: Woodley (2001)]

**Local distribution and dates of collection** (Fig. 11): EGYPT: Coastal Strip: Mersa Matrouh (July and August). [Source: original description (Mohammad et al. (2009)]

##### Notes

**Figure 12.**
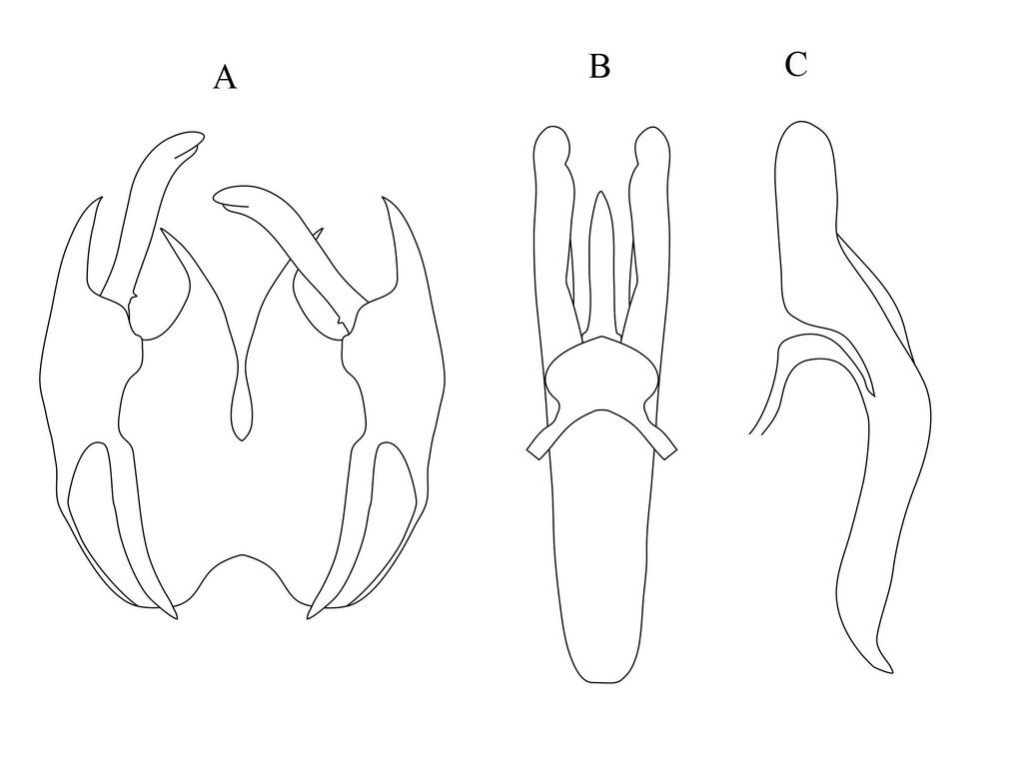
*Nemotelus matrouhensis* Mohammad et al.: **A.** Male terminalia, dorsal view; **B.** Phallic complex, dorsal view; **C.** same, lateral view.

**Figure 13.**
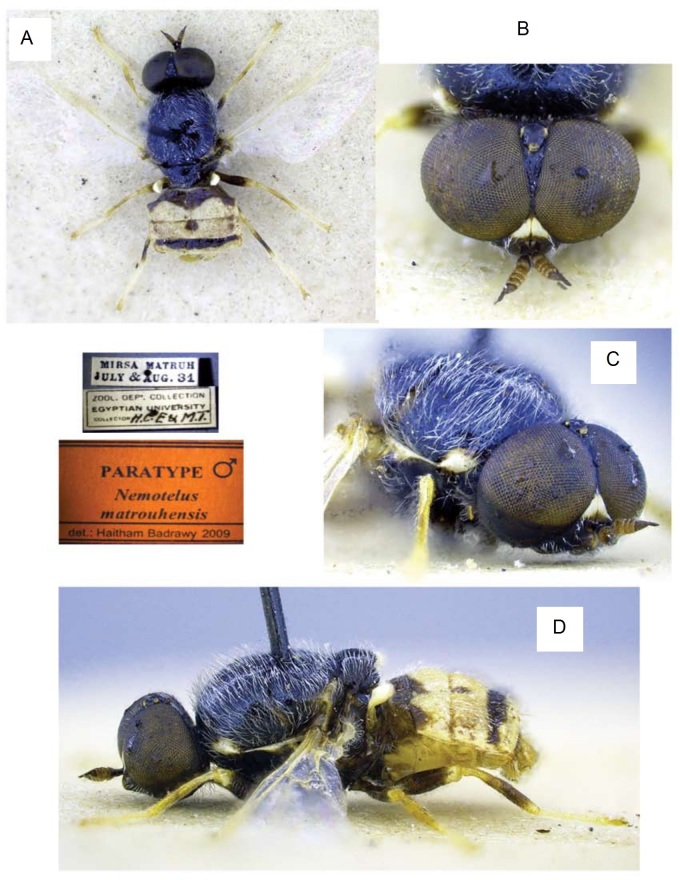
*Nemotelus matrouhensis* Mohammad et al.: **A.** Male habitus, dorsal view; **B.** Head, frontal view; **C.** Head and thorax, frontolateral view; **D.** Male habitus, lateral view.

Mohammad et al. (2009) published this species and inaccurately cited 13 males and 8 females as "holotype". They did not explicitly select a single specimen as holotype, so all cited specimens are considered as syntypes and a lectotype should be designated. As the illustration of the male genitalia was made from an intact specimen and did not show all the important structures, we are providing an illustration of dissected genitalia (Fig. 12) and a habitus photograph of the specimen (Fig. 13). When one of us (MH) requested a specimen, we were told that this would be a paratype, but because the specimen had no identification or type label at all, a red label was generated and attached to this specimen. This label reads: Paratype ♂/ *Nemotelus matrouhensis*/ det.: Haitham Badrawy 2009. However, now it is clear that none of the specimens had identification labels and also that this was not a paratype, but a syntype. The male genitalia resemble *N. crenatus* Egger, 1859 and *N. obscuripes* Loew, 1871. Lectotype designation: a male specimen in EFC is herein designated as lectotype and is labelled: Mirsa Matruh, July & Aug 1931 (leg. H.C.E. & M.T.), with a red lectotype label. Paralectotypes: 12 males and 8 females, same data.

#### Nemotelus (Nemotelus) niloticus

Olivier, 1811

E355FEE9-6CA4-509F-B3B7-A142D0634414

https://www.gbif.org/species/1578794

Nemotelus niloticus Olivier, 1811: 183. Type locality: Egypt.
Nemotelus fasciatus Olivier, 1811: 183. Type locality: Egypt (“bords du Nil & des canaux qui en dérivent”). Preoccupied by *Nemotelus fasciatus* Geoffroy in Fourcroy, 1785.
Nemotelus albifacies Becker, 1902: 9. Type locality: Egypt (Alexandria).
Nemotelus oasis Becker, 1906: 6. Type locality: Algeria (Biskra).
Nemotelus theodori Lindner, 1974: 95. Type locality: Israel (Arava Valley: Hazeva).
Nemotelus duofasciatus Woodley 2001. Replacement name for *N. fasciatus* Olivier, 1811.

##### Materials

- **Type status:** Other material. **Occurrence:** recordedBy: Efflatoun; lifeStage: Adult; **Taxon:** taxonID: https://www.gbif.org/species/1578794; scientificName: Nemotelus niloticus; **Location:** country: Egypt; locality: Fayoum City; decimalLatitude: 29.32061; decimalLongitude: 30.818; **Identification:** identifiedBy: M. El-Hawagry & M. Hauser; dateIdentified: 2020-2021; **Event:** samplingProtocol: Not given; eventDate: 05-12-1918; **Record Level:** institutionCode: ESEC
- **Type status:** Other material. **Occurrence:** recordedBy: Efflatoun; lifeStage: Adult; **Taxon:** taxonID: https://www.gbif.org/species/1578794; scientificName: Nemotelus niloticus; **Location:** country: Egypt; locality: Beheira; decimalLatitude: 30.62189; decimalLongitude: 30.48755; **Identification:** identifiedBy: M. El-Hawagry & M. Hauser; dateIdentified: 2020-2021; **Event:** samplingProtocol: Not given; eventDate: Apr 1924; **Record Level:** institutionCode: ESEC
- **Type status:** Other material. **Occurrence:** recordedBy: Efflatoun; lifeStage: Adult; **Taxon:** taxonID: https://www.gbif.org/species/1578794; scientificName: Nemotelus niloticus; **Location:** country: Egypt; locality: Damietta; decimalLatitude: 31.34595; decimalLongitude: 31.6317; **Identification:** identifiedBy: M. El-Hawagry & M. Hauser; dateIdentified: 2020-2021; **Event:** samplingProtocol: Not given; eventDate: 05-11-1918; **Record Level:** institutionCode: ESEC
- **Type status:** Other material. **Occurrence:** recordedBy: Efflatoun; lifeStage: Adult; **Taxon:** taxonID: https://www.gbif.org/species/1578794; scientificName: Nemotelus niloticus; **Location:** country: Egypt; locality: Quisna; decimalLatitude: 30.53514; decimalLongitude: 31.1117; **Identification:** identifiedBy: M. El-Hawagry & M. Hauser; dateIdentified: 2020-2021; **Event:** samplingProtocol: Not given; eventDate: Mar 1924; **Record Level:** institutionCode: ESEC
- **Type status:** Other material. **Occurrence:** recordedBy: Tewfik; sex: 1 female; lifeStage: Adult; **Taxon:** taxonID: https://www.gbif.org/species/1578794; scientificName: Nemotelus niloticus; **Location:** country: Egypt; locality: Mariout; decimalLatitude: 31.0172; decimalLongitude: 29.76; **Identification:** identifiedBy: M. El-Hawagry & M. Hauser; dateIdentified: 2020-2021; **Event:** samplingProtocol: Not given; eventDate: 07-09-1927; **Record Level:** institutionCode: EFC
- **Type status:** Other material. **Occurrence:** recordedBy: Efflatoun; sex: 4 males, 11 females; lifeStage: Adult; **Taxon:** taxonID: https://www.gbif.org/species/1578794; scientificName: Nemotelus niloticus; **Location:** country: Egypt; locality: Mariout (Mallaha); decimalLatitude: 31.0172; decimalLongitude: 29.76; **Identification:** identifiedBy: M. El-Hawagry & M. Hauser; dateIdentified: 2020-2021; **Event:** samplingProtocol: Not given; eventDate: 06-16-1929; **Record Level:** institutionCode: EFC
- **Type status:** Other material. **Occurrence:** recordedBy: Efflatoun; sex: 2 males; lifeStage: Adult; **Taxon:** taxonID: https://www.gbif.org/species/1578794; scientificName: Nemotelus niloticus; **Location:** country: Egypt; locality: Fayoum City; decimalLatitude: 29.32061; decimalLongitude: 30.818; **Identification:** identifiedBy: M. El-Hawagry & M. Hauser; dateIdentified: 2020-2021; **Event:** samplingProtocol: Not given; eventDate: 04-23-1943; **Record Level:** institutionCode: EFC
- **Type status:** Other material. **Occurrence:** recordedBy: Efflatoun; sex: 2 females; lifeStage: Adult; **Taxon:** taxonID: https://www.gbif.org/species/1578794; scientificName: Nemotelus niloticus; **Location:** country: Egypt; locality: Fayoum City; decimalLatitude: 29.32061; decimalLongitude: 30.818; **Identification:** identifiedBy: M. El-Hawagry & M. Hauser; dateIdentified: 2020-2021; **Event:** samplingProtocol: Not given; eventDate: 04-20-1945; **Record Level:** institutionCode: EFC
- **Type status:** Other material. **Occurrence:** recordedBy: Efflatoun; sex: 1 female; lifeStage: Adult; **Taxon:** taxonID: https://www.gbif.org/species/1578794; scientificName: Nemotelus niloticus; **Location:** country: Egypt; locality: Giza-Fayoum Road; decimalLatitude: 29.5564; decimalLongitude: 30.8869; **Identification:** identifiedBy: M. El-Hawagry & M. Hauser; dateIdentified: 2020-2021; **Event:** samplingProtocol: Not given; eventDate: 04-14-1947; **Record Level:** institutionCode: EFC
- **Type status:** Other material. **Occurrence:** recordedBy: Farag; sex: 1 male; lifeStage: Adult; **Taxon:** taxonID: https://www.gbif.org/species/1578794; scientificName: Nemotelus niloticus; **Location:** country: Egypt; locality: Helwan; decimalLatitude: 29.8500; decimalLongitude: 31.3333; **Identification:** identifiedBy: M. El-Hawagry & M. Hauser; dateIdentified: 2020-2021; **Event:** samplingProtocol: Not given; eventDate: 04-08-1934; **Record Level:** institutionCode: EFC
- **Type status:** Other material. **Occurrence:** recordedBy: Farag; sex: 1 female; lifeStage: Adult; **Taxon:** taxonID: https://www.gbif.org/species/1578794; scientificName: Nemotelus niloticus; **Location:** country: Egypt; locality: Helwan; decimalLatitude: 29.8500; decimalLongitude: 31.3333; **Identification:** identifiedBy: M. El-Hawagry & M. Hauser; dateIdentified: 2020-2021; **Event:** samplingProtocol: Not given; eventDate: 04-14-1934; **Record Level:** institutionCode: EFC
- **Type status:** Other material. **Occurrence:** recordedBy: Farag; sex: 1 female; lifeStage: Adult; **Taxon:** taxonID: https://www.gbif.org/species/1578794; scientificName: Nemotelus niloticus; **Location:** country: Egypt; locality: Helwan; decimalLatitude: 29.8500; decimalLongitude: 31.3333; **Identification:** identifiedBy: M. El-Hawagry & M. Hauser; dateIdentified: 2020-2021; **Event:** samplingProtocol: Not given; eventDate: 04-24-1934; **Record Level:** institutionCode: EFC
- **Type status:** Other material. **Occurrence:** recordedBy: Farag; sex: 1 female; lifeStage: Adult; **Taxon:** taxonID: https://www.gbif.org/species/1578794; scientificName: Nemotelus niloticus; **Location:** country: Egypt; locality: WadiHoff; decimalLatitude: 29.8821; decimalLongitude: 31.311; **Identification:** identifiedBy: M. El-Hawagry & M. Hauser; dateIdentified: 2020-2021; **Event:** samplingProtocol: Not given; eventDate: 04-04-1930; **Record Level:** institutionCode: EFC
- **Type status:** Other material. **Occurrence:** recordedBy: Efflatoun; sex: 1 female; lifeStage: Adult; **Taxon:** taxonID: https://www.gbif.org/species/1578794; scientificName: Nemotelus niloticus; **Location:** country: Egypt; locality: Ramleh; decimalLatitude: 31.2279; decimalLongitude: 29.976; **Identification:** identifiedBy: M. El-Hawagry & M. Hauser; dateIdentified: 2020-2021; **Event:** samplingProtocol: Not given; eventDate: 09-17-1921; **Record Level:** institutionCode: EFC
- **Type status:** Other material. **Occurrence:** recordedBy: Efflatoun; sex: 1 male; lifeStage: Adult; **Taxon:** taxonID: https://www.gbif.org/species/1578794; scientificName: Nemotelus niloticus; **Location:** country: Egypt; locality: Dekhela; decimalLatitude: 31.12098; decimalLongitude: 29.8156; **Identification:** identifiedBy: M. El-Hawagry & M. Hauser; dateIdentified: 2020-2021; **Event:** samplingProtocol: Not given; eventDate: 06-24-1926; **Record Level:** institutionCode: EFC
- **Type status:** Other material. **Occurrence:** recordedBy: H.C.E & M.T.; sex: 1 female; lifeStage: Adult; **Taxon:** taxonID: https://www.gbif.org/species/1578794; scientificName: Nemotelus niloticus; **Location:** country: Egypt; locality: Dekhela; decimalLatitude: 31.12098; decimalLongitude: 29.8156; **Identification:** identifiedBy: M. El-Hawagry & M. Hauser; dateIdentified: 2020-2021; **Event:** samplingProtocol: Not given; eventDate: 06-24-1926; **Record Level:** institutionCode: EFC
- **Type status:** Other material. **Occurrence:** recordedBy: Efflatoun; sex: 1 male; lifeStage: Adult; **Taxon:** taxonID: https://www.gbif.org/species/1578794; scientificName: Nemotelus niloticus; **Location:** country: Egypt; locality: Dekhela; decimalLatitude: 31.12098; decimalLongitude: 29.8156; **Identification:** identifiedBy: M. El-Hawagry & M. Hauser; dateIdentified: 2020-2021; **Event:** samplingProtocol: Not given; eventDate: 06-18-1929; **Record Level:** institutionCode: EFC
- **Type status:** Other material. **Occurrence:** recordedBy: Efflatoun; sex: 1 male; lifeStage: Adult; **Taxon:** taxonID: https://www.gbif.org/species/1578794; scientificName: Nemotelus niloticus; **Location:** country: Egypt; locality: Ismaila; decimalLatitude: 30.32382; decimalLongitude: 32.3008; **Identification:** identifiedBy: M. El-Hawagry & M. Hauser; dateIdentified: 2020-2021; **Event:** samplingProtocol: Not given; eventDate: 10-14-1926; **Record Level:** institutionCode: EFC

##### Distribution

AF: United Arab Emirates. PA: Algeria, Egypt, Israel, Italy (Sardinia), Tunisia. [Sources: Woodley (2001), Mason et al. (2009) and Hauser (2008)]

**Figure 14.**
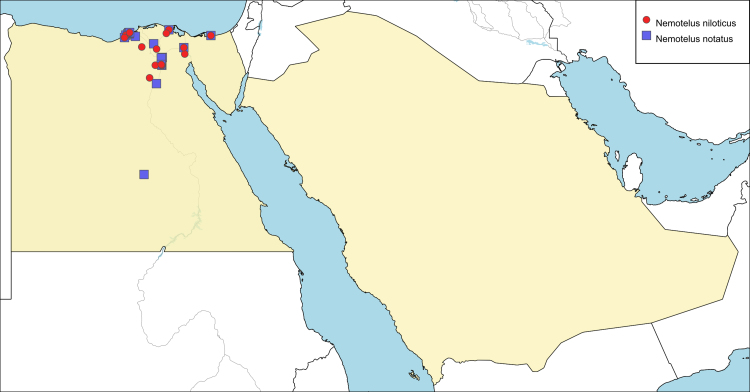
Distribution map of *Nemotelus niloticus* Olivier and *Nemotelus notatus* Zetterstedt.

**Local distribution and dates of collection** (Fig. 14): EGYPT: Coastal Strip: Alexandria, Dekhela, Mariout (Mallaha), Ramleh (May to September). Eastern Desert: Fayed, Ismailia, Wadi Hoff (April, May and October). Fayoum: Fayoum City, Giza-Fayoum Road (April and May). Lower Nile Valley and Delta: Beheira, Damietta, Helwan, Quisna, Sherbin (March to September). Sinai: Zaranik Protectorate (April). [Sources: original description of *N. albifacies* (Becker 1902, Mohammad et al. (2009) and museum material]

#### Nemotelus (Nemotelus) notatus

Zetterstedt, 1842

B981BA33-3E05-57CF-B384-522534F45E6C

https://www.gbif.org/species/1578742

Nemotelus notatus Zetterstedt, 1842: 148. Type locality: Denmark (Copenhagen).
Nemotelus brachystomus Loew, 1846: 443. Type locality: Croatia (as “Dalmatien”).
Nemotelus leucorhynchus A. Costa, 1884: 61. Type locality: Italy (Sardinia: Stagno di Caliari).
Nemotelus nigroaeneus Verhoeff, 1891: 3. Type locality: Germany (Insel Norderney).
Nemotelus punctiventris Becker, 1902: 8. Type locality: Egypt (Alexandria).
Nemotelus brachystomus form aegyptiacus Lindner, 1925: 146. Type locality: Egypt (Alexandria: Nuzha, Cleopatra, Moharrem Bey, and Aboukir).
Nemotelus nigroaeneus ab. portalis Szilády, 1932: 33. Type locality: Germany (Borkum).
Nemotelus balearicus Lindner, 1937: 121. Type locality: Spain (Balearic Islands).
Nemotelus zernyi Lindner, 1937: 147. Type locality: Spain (Andalucía: Algeciras).

##### Materials

- **Type status:** Other material. **Occurrence:** recordedBy: Efflatoun; lifeStage: Adult; **Taxon:** taxonID: https://www.gbif.org/species/1578742; scientificName: Nemotelus notatus; **Location:** country: Egypt; locality: Tanta; decimalLatitude: 30.75725; decimalLongitude: 30.9898; **Identification:** identifiedBy: M. El-Hawagry & M. Hauser; dateIdentified: 2020-2021; **Event:** samplingProtocol: Not given; eventDate: 05-11-1918; **Record Level:** institutionCode: ESEC
- **Type status:** Other material. **Occurrence:** recordedBy: Efflatoun; lifeStage: Adult; **Taxon:** taxonID: https://www.gbif.org/species/1578742; scientificName: Nemotelus notatus; **Location:** country: Egypt; locality: Khusus; decimalLatitude: 30.15957; decimalLongitude: 31.3125; **Identification:** identifiedBy: M. El-Hawagry & M. Hauser; dateIdentified: 2020-2021; **Event:** samplingProtocol: Not given; eventDate: Apr 1918; **Record Level:** institutionCode: ESEC
- **Type status:** Other material. **Occurrence:** recordedBy: Efflatoun; lifeStage: Adult; **Taxon:** taxonID: https://www.gbif.org/species/1578742; scientificName: Nemotelus notatus; **Location:** country: Egypt; locality: Alag; decimalLatitude: 30.18009; decimalLongitude: 31.3521; **Identification:** identifiedBy: M. El-Hawagry & M. Hauser; dateIdentified: 2020-2021; **Event:** samplingProtocol: Not given; eventDate: Nov 1924; **Record Level:** institutionCode: ESEC
- **Type status:** Other material. **Occurrence:** recordedBy: Efflatoun; lifeStage: Adult; **Taxon:** taxonID: https://www.gbif.org/species/1578742; scientificName: Nemotelus notatus; **Location:** country: Egypt; locality: Marg; decimalLatitude: 31.0667; decimalLongitude: 30.2167; **Identification:** identifiedBy: M. El-Hawagry & M. Hauser; dateIdentified: 2020-2021; **Event:** samplingProtocol: Not given; eventDate: Mar 1918; **Record Level:** institutionCode: ESEC
- **Type status:** Other material. **Occurrence:** recordedBy: Efflatoun; lifeStage: Adult; **Taxon:** taxonID: https://www.gbif.org/species/1578742; scientificName: Nemotelus notatus; **Location:** country: Egypt; locality: El-Mahareeq (Kharga); decimalLatitude: 25.618206; decimalLongitude: 30.6468; **Identification:** identifiedBy: M. El-Hawagry & M. Hauser; dateIdentified: 2020-2021; **Event:** samplingProtocol: Not given; eventDate: Feb 1914; **Record Level:** institutionCode: ESEC
- **Type status:** Other material. **Occurrence:** recordedBy: Efflatoun; lifeStage: Adult; **Taxon:** taxonID: https://www.gbif.org/species/1578742; scientificName: Nemotelus notatus; **Location:** country: Egypt; locality: Beni Sweif; decimalLatitude: 29.07788; decimalLongitude: 31.10713; **Identification:** identifiedBy: M. El-Hawagry & M. Hauser; dateIdentified: 2020-2021; **Event:** samplingProtocol: Not given; eventDate: 02-14-1914; **Record Level:** institutionCode: ESEC
- **Type status:** Other material. **Occurrence:** recordedBy: Efflatoun; lifeStage: Adult; **Taxon:** taxonID: https://www.gbif.org/species/1578742; scientificName: Nemotelus notatus; **Location:** country: Egypt; locality: Kharga Oasis; decimalLatitude: 25.25; decimalLongitude: 30.5833; **Identification:** identifiedBy: M. El-Hawagry & M. Hauser; dateIdentified: 2020-2021; **Event:** samplingProtocol: Not given; eventDate: Feb 1914; **Record Level:** institutionCode: ESEC
- **Type status:** Other material. **Occurrence:** recordedBy: Priesner; lifeStage: Adult; **Taxon:** taxonID: https://www.gbif.org/species/1578742; scientificName: Nemotelus notatus; **Location:** country: Egypt; locality: Kharga Oasis; decimalLatitude: 25.25; decimalLongitude: 30.5833; **Identification:** identifiedBy: M. El-Hawagry & M. Hauser; dateIdentified: 2020-2021; **Event:** samplingProtocol: Not given; eventDate: 03-10-1924; **Record Level:** institutionCode: ESEC
- **Type status:** Other material. **Occurrence:** recordedBy: Efflatoun; sex: 1 female; lifeStage: Adult; **Taxon:** taxonID: https://www.gbif.org/species/1578742; scientificName: Nemotelus notatus; **Location:** country: Egypt; locality: Cleopatra; decimalLatitude: 31.22021; decimalLongitude: 29.9348; **Identification:** identifiedBy: M. El-Hawagry & M. Hauser; dateIdentified: 2020-2021; **Event:** samplingProtocol: Not given; eventDate: 07-12-1921; **Record Level:** institutionCode: EFC
- **Type status:** Other material. **Occurrence:** recordedBy: H.C.E & M.T.; sex: 1 male; lifeStage: Adult; **Taxon:** taxonID: https://www.gbif.org/species/1578742; scientificName: Nemotelus notatus; **Location:** country: Egypt; locality: Dekhela; decimalLatitude: 31.12098; decimalLongitude: 29.8156; **Identification:** identifiedBy: M. El-Hawagry & M. Hauser; dateIdentified: 2020-2021; **Event:** samplingProtocol: Not given; eventDate: 06-24-1926; **Record Level:** institutionCode: EFC
- **Type status:** Other material. **Occurrence:** recordedBy: H.C.E & M.T.; sex: 3 males, 5 females; lifeStage: Adult; **Taxon:** taxonID: https://www.gbif.org/species/1578742; scientificName: Nemotelus notatus; **Location:** country: Egypt; locality: Dekhela; decimalLatitude: 31.12098; decimalLongitude: 29.8156; **Identification:** identifiedBy: M. El-Hawagry & M. Hauser; dateIdentified: 2020-2021; **Event:** samplingProtocol: Not given; eventDate: 05-24-1925; **Record Level:** institutionCode: EFC
- **Type status:** Other material. **Occurrence:** recordedBy: Efflatoun; sex: 1 male, 1 female; lifeStage: Adult; **Taxon:** taxonID: https://www.gbif.org/species/1578742; scientificName: Nemotelus notatus; **Location:** country: Egypt; locality: Dekhela; decimalLatitude: 31.12098; decimalLongitude: 29.8156; **Identification:** identifiedBy: M. El-Hawagry & M. Hauser; dateIdentified: 2020-2021; **Event:** samplingProtocol: Not given; eventDate: 07-09-1927; **Record Level:** institutionCode: EFC
- **Type status:** Other material. **Occurrence:** sex: 1 female; lifeStage: Adult; **Taxon:** taxonID: https://www.gbif.org/species/1578742; scientificName: Nemotelus notatus; **Location:** country: Egypt; locality: Fayed; decimalLatitude: 30.32382; decimalLongitude: 32.3008; **Identification:** identifiedBy: M. El-Hawagry & M. Hauser; dateIdentified: 2020-2021; **Event:** samplingProtocol: Not given; eventDate: 07-24-1924; **Record Level:** institutionCode: EFC
- **Type status:** Other material. **Occurrence:** recordedBy: Farag; sex: ?; lifeStage: Adult; **Taxon:** taxonID: https://www.gbif.org/species/1578742; scientificName: Nemotelus notatus; **Location:** country: Egypt; locality: Helwan; decimalLatitude: 29.8500; decimalLongitude: 31.3333; **Identification:** identifiedBy: M. El-Hawagry & M. Hauser; dateIdentified: 2020-2021; **Event:** samplingProtocol: Not given; eventDate: 04-08-1934; **Record Level:** institutionCode: EFC
- **Type status:** Other material. **Occurrence:** recordedBy: Tewfik; sex: ?; lifeStage: Adult; **Taxon:** taxonID: https://www.gbif.org/species/1578742; scientificName: Nemotelus notatus; **Location:** country: Egypt; locality: Wadi Hoff; decimalLatitude: 29.8821; decimalLongitude: 31.311; **Identification:** identifiedBy: M. El-Hawagry & M. Hauser; dateIdentified: 2020-2021; **Event:** samplingProtocol: Not given; eventDate: 06-09-1927; **Record Level:** institutionCode: EFC

##### Distribution

PA: Albania, Austria, Azerbaijan, Belgium, Bulgaria, Croatia, Cyprus, Denmark, Egypt, England, Finland, France, Germany, Greece, Hungary, Iran, Ireland, Israel, Italy (including Sardinia), Netherlands, Norway, Poland, Romania, Russia, Spain, Sweden, Turkey, former Yugoslavia. [Sources: Woodley (2001), Üstüner and Hasbenli (2005), Mason et al. (2009) and Khaghaninia et al. (2015)]

**Local distribution and dates of collection** (Fig. 14): EGYPT: Coastal Strip: Abu-Kir, Cleopatra, Dekhela, Mariout, Moharram Bey, Nubar Bey, Nuzha, Ramleh (April to September). Eastern Desert: Ismailia, Wadi Hoff (May to July). Lower Nile Valley and Delta: Beni Sweif, Damietta, El-Alag, Helwan, El-Marg, Khusous, Tanta (February to May and November). Sinai: Zaranik Protectorate (April). Western Desert: Kharga Oasis (February and March). [Sources: original description of *N. b. aegyptiacus* (Lindner 1925), Mohammad et al. (2009) and museum material]

## Supplementary Material

XML Treatment for Stratiomyidae

XML Treatment for Stratiomyiinae

XML Treatment for Aspidacantha

XML Treatment for Aspidacantha atra

XML Treatment for Sternobrithes

XML Treatment for Sternobrithes sp.

XML Treatment for Stratiomyinae

XML Treatment for Oxycerini

XML Treatment for Oxycera

XML Treatment for Oxycera orientalis

XML Treatment for Oxycera turcica

XML Treatment for Stratiomyini

XML Treatment for Odontomyia

XML Treatment for Odontomyia angulata

XML Treatment for Odontomyia megacephala

XML Treatment for Odontomyia xanthopus

XML Treatment for Oplodontha

XML Treatment for Oplodontha minuta

XML Treatment for Oplodontha pulchriceps

XML Treatment for Stratiomys

XML Treatment for Stratiomys cenisia

XML Treatment for Stratiomys deserticolor

XML Treatment for Stratiomys longicornis

XML Treatment for Stratiomys singularior

XML Treatment for Nemotelinae

XML Treatment for Nemotelus

XML Treatment for Nemotelus

XML Treatment for Nemotelus (Nemotelus) anchora

XML Treatment for Nemotelus (Nemotelus) candidus

XML Treatment for Nemotelus (Nemotelus) dentatus

XML Treatment for Nemotelus (Nemotelus) marinus

XML Treatment for Nemotelus (Nemotelus) matrouhensis

XML Treatment for Nemotelus (Nemotelus) niloticus

XML Treatment for Nemotelus (Nemotelus) notatus

## Figures and Tables

**Table 1.** A gazetteer of Egyptian and Saudi Arabian localities of the family Stratiomyidae.

**Country**	**Locality**	**Governorate**	**Ecological zone or Region**	**Latitude (N)**	**Longitude (E)**
Egypt	Abu-Kir	Alexandria	Coastal Strip	31.22429	33.8256
	Abu-Rawash	Giza	Lower Nile Valley & Delta	30.0438	42.4101
	Abu-Zaabal	Al-Qalyubia	Lower Nile Valley & Delta	30.24098	42.4089
	Alexandria	Alexandria	Coastal Strip	31.2129	42.4089
	Behaira	Behaira	Lower Nile Valley & Delta	30.62189	42.4102
	Benha	Al-Qalyubia	Lower Nile Valley & Delta	30.46572	42.4089
	Beni Sweif	Beni Sweif	Lower Nile Valley & Delta	29.07788	42.4089
	Birket Qaroun	Fayoum	Fayoum	29.40879	42.4124
	Cleopatra	Alexandria	Coastal Strip	31.22022	42.4101
	Dakhla Oasis	New Valley	Western Desert	25.5000	41.3115
	Damietta	Damietta	Lower Nile Valley & Delta	31.34595	41.3115
	Dekhela	Alexandria	Coastal Strip	31.12098	41.3101
	Ein Moussa	South Sinai	Sinai	29.8667	41.3101
	El-Alag	Al-Qalyubia	Lower Nile Valley & Delta	30.18009	42.8348
	El-Arish	North Sinai	Sinai	31.1244	39.8749
	El-Baragil	Giza	Lower Nile Valley & Delta	30.07673	41.4107
	El-Ferdan	Ismailia	Eastern Desert	30.65760	36.6297
	El-Gebel El-Asfar	Al-Qalyubia	Lower Nile Valley & Delta	29.1689	34.1412
	El-Kantara	Ismailia	Eastern Desert	30.79392	34.3836
	El-Marg	Al-Qalyubia	Lower Nile Valley & Delta	31.0667	30.5833
	El-Siala	Alexandria	Coastal Strip	31.20849	30.8180
	El-Tour	South Sinai	Sinai	28.24024	29.1667
	Ezbet El-Nakhl	Al-Qalyubia	Lower Nile Valley & Delta	31.1111	32.6500
	Fayed	Ismailia	Eastern Desert	30.32382	29.8805
	Fayoum	Fayoum	Fayoum	29.32061	32.3008
	Gebel Elba	Red Sea	Gebel Elba	22.2008	30.2167
	Gezeira	Cairo	Lower Nile Valley & Delta	30.04596	34.3836
	Girza	Fayoum	Fayoum	29.49968	34.1412
	Giza	Giza	Lower Nile Valley & Delta	30.01350	31.3333
	Giza-Fayoum Road	Giza	Western Desert	29.85176	31.18121
	Helwan	Cairo	Lower Nile Valley & Delta	29.8500	31.22435
	Hurghada	Red Sea	Eastern Desert	27.2337	30.5833
	Ismailia	Ismailia	Eastern Desert	30.59428	30.8180
	Kerdassa	Giza	Lower Nile Valley & Delta	30.0297	30.48755
	Kharga Oasis	New Valley	Western Desert	25.2500	31.6317
	Khosous	Cairo	Lower Nile Valley & Delta	30.15957	31.1117
	Kosseimah	North Sinai	Sinai	30.90307	30.9898
	Maadi	Cairo	Lower Nile Valley & Delta	29.95772	31.3125
	Mariout	Alexandria	Coastal Strip	31.0172	31.3531
	Max	Alexandria	Coastal Strip	31.1636	30.2167
	Mersa Matrouh	Matrouh	Coastal Strip	29.5696	30.6468
	Moharram Bey	Alexandria	Coastal Strip	31.17796	31.10713
	Moweileh	South Sinai	Sinai	30.3924	30.5833
	Nazla	Fayoum	Fayoum	29.29792	30.5833
	Nubar Bey	Alexandria	Coastal Strip	31.18079	31.2505
	Nuzha (Alex.)	Alexandria	Coastal Strip	31.2001	31.3333
	Quisna	Menofiya	Lower Nile Valley & Delta	30.53514	31.3333
	Ramleh	Alexandria	Coastal Strip	31.2279	30.8180
	Sandoub	Dakahlyia	Lower Nile Valley & Delta	31.00782	30.2167
	Sherbin	Dakahlyia	Lower Nile Valley & Delta	31.19461	31.3333
	Shubra	Al-Qalyoubia	Lower Nile Valley & Delta	30.1012	30.5833
	Siwa Oasis	Matrouh	Western Desert	29.20427	26.4194
	Suez	Suez	Eastern Desert	29.95278	32.3008
	Tanta	Gharbia	Lower Nile Valley & Delta	30.75725	32.3008
	Wadi El-Lega	South Sinai	Sinai	28.5469	32.3008
	Wadi El-Natroun	Behaira	Western Desert	30.3814	29.8157
	Wadi Hebran	South Sinai	Sinai	28.40225	31.3521
	Wadi Hoff	Cairo	Eastern Desert	29.8821	32.3008
	Wadi Watir	South Sinai	Sinai	29.02147	31.3110
	Zaranik	North Sinai	Sinai	31.10345	30.3441
Saudi Arabia	Al-Mekhwa	Al-Mekhwa	Al-Baha	19.798133	30.3441
	Dawmat Al-Jandal	Dawmat Al-Gandal	Al-Jwaf	29.809552	30.8180
	Hassan Ameen farm	Tabouk	Tabouk	28.36661	26.4194
	Jabal Shada al-A’la Nature Reserve	Al-Mekhwa	Al-Baha	19.8429	29.7600
	Jazan	Jazan	Jazan	16.9595	29.7600
	Raydah Nature Reserve	Abha	Asir	18.20525	30.8180
